# Serologic response and safety of COVID-19 vaccination in HSCT or CAR T-cell recipients: a systematic review and meta-analysis

**DOI:** 10.1186/s40164-022-00299-6

**Published:** 2022-08-16

**Authors:** Chenghao Ge, Kelei Du, Mingjie Luo, Kaini Shen, Yangzhong Zhou, Kaiyuan Guo, Yang Liu, Chen Yin, Yi Li, Guanqiao Li, Xiaoyuan Chen

**Affiliations:** 1 Tsinghua Clinical Research Institute, School of Medicine, Tsinghua University, Beijing, China; 2 School of Medicine, Tsinghua University, Beijing, China; 3 Department of Hematology, Peking Union Medical College Hospital, Chinese Academy of Medical Sciences & Peking Union Medical College, Beijing, China; 4 Department of Rheumatology and Clinical Immunology, Peking Union Medical College Hospital, Chinese Academy of Medical Sciences & Peking Union Medical College, Beijing, China; 5 Vanke School of Public Health, Tsinghua University, Beijing, China; 6 Office of Clinical Trial Institute, Beijing Tsinghua Changgung Hospital, Beijing, China

**Keywords:** COVID-19, Vaccine, Hematopoietic stem cell transplantation, Chimeric antigen receptor T cell therapy, Meta-analysis

## Abstract

**Background:**

Patients receiving hematopoietic stem cell transplantation (HSCT) or chimeric antigen receptor T cell (CAR T-cell) therapy are immunocompromised and at high risk of viral infection, including SAR2-CoV-2 infection. However, the effectiveness and safety of COVID-19 vaccines in these recipients is not well characterized. The present meta-analysis evaluated the serologic response and safety of COVID-19 vaccines in these population.

**Methods:**

Literature databases (MEDLINE, EMBASE, Web of Science, MedRvix and BioRvix) were searched for original studies with serologic response post COVID-19 vaccination in HSCT or CAR T-cell recipients published until July 14, 2022. The analysis included 27 observational studies with a total of 2899 patients receiving allogeneic HSCT (2506), autologous HSCT (286) or CAR T-cell therapy (107), and 683 healthy participants with serologic response data. Random effects models were used to pool the rate of serologic response to COVID-19 vaccination in HSCT or CAR T-cell recipients and odds ratio comparing with healthy controls.

**Results:**

The pooled seropositivity rates in HSCT and CAR T-cell recipients were 0.624 [0.506–0.729] for one dose, 0.745 [0.712–0.776] for two doses. The rates were significantly lower than those in healthy controls (nearly 100%). In subgroup analysis, CAR T-cell recipients exhibited an even lower seroconversion rate (one dose: 0.204 [0.094–0.386]; two doses: 0.277 [0.190–0.386]) than HSCT counterparts (one dose: 0.779 [0.666–0.862]; two doses: 0.793 [0.762–0.821]). The rates were comparable between autologous and allogeneic HSCT recipients. Other possible impact factors related to seropositivity were time interval between therapy and vaccination, use of immunosuppressive drugs and immune cell counts. Most vaccine-related adverse effects were mild and resolvable, comparable to general population.

**Conclusions:**

This analysis revealed a diminished response to COVID-19 vaccines in HSCT or CAR T-cell recipients. Our findings may inform regular COVID-19 vaccination at appropriate intervals after HSCT or CAR T-cell therapy.

**Supplementary Information:**

The online version contains supplementary material available at 10.1186/s40164-022-00299-6.

## Introduction

Since the outbreak of the human coronavirus disease 2019 (COVID-19), the pandemic has posed tremendous challenges to the globe, with many new variants emerging. According to the World Health Organization (WHO), as of July 15, 2022, the cumulative number of confirmed COVID-19 cases worldwide reached over 557 million and the cumulative number of deaths reached over 6.35 million [1]. In response, many countries have adopted mass COVID-19 vaccination and booster shot programs [2, 3]. The COVID-19 vaccines have been validated in large-scale clinical trials and real-world settings to be protective and well-tolerable in general populations [4, 5]. However, the effectiveness and safety of COVID-19 vaccines in specific populations, such as immunosuppressed patients and patients with cancer, have not been well characterized, given that they were usually excluded from registrational clinical trials or defined as “warnings and precautions” groups in the labels [6, 7].

Hematopoietic stem cell transplantation (HSCT) and chimeric antigen receptor T cell (CAR T-cell) have been standard-of-care or emerging treatment options for multiple diseases, such as hematological malignancies and autoimmune diseases, which are featured by dysfunction of hematopoietic or immune system [8–10]. Basically, HSCT involves depleting recipients’ dysfunctional hematopoietic and immune system and infusing autologous or allogeneic stem cells to achieve immune reconstitution, so-called autologous HSCT (auto-HSCT) or allogeneic HSCT (allo-HSCT), respectively [11–13]. For CAR T-cell therapy, either autologous or allogeneic T cells separated from peripheral blood are manufactured to target specific antigens and transfused back to patients after the ablative chemotherapy. Globally, as of April 2022, five allogeneic CD19-directed CAR T-cell [14] and two allogeneic B-cell maturation antigen (BMCA)-oriented CAR T-cell products have been approved for B-cell malignancies and multiple myeloma, respectively [15]. HSCT or CAR T-cell recipients are generally immunocompromised and vulnerable to infection post-transplantation due to underlying diseases, depletive conditioning regimens, multiple immunosuppressive treatments, or long-term application of immunosuppressants post-therapy [16, 17]. Once infected with SARS-CoV-2, they are prone to developing severe or fatal symptoms, associated with higher rates of hospitalization and mortality [18, 19]. On this basis, the European and US transplant guidelines consider that the benefits of COVID-19 vaccination may overweigh risks for patients receiving HSCT or CAR T-cell and recommend COVID-19 vaccination as early as three months after transplantation or cell therapy [20, 21]. These recommendations were based on limited evidence from individual studies on COVID-19 vaccines with small sample size and previous clinical experience with infections caused by other pathogens. Therefore, it is imperative to integrate findings across studies to gain a better understanding into the effectiveness of COVID-19 vaccines in this population. In this study, we systematically evaluated the serologic responses and safety after COVID-19 vaccination in patients receiving HSCT or CAR T-cell therapy and aimed at providing insights on COVID-19 vaccination programs for this population.

## Methods

This meta-analysis was conducted according to the preferred reporting items for systematic reviews and meta-analyses (PRISMA) guidelines, and the PRISMA checklist is available in Additional file 1: Table S1. This study was registered at the International Prospective Register of Systematic Reviews (PROSPERO) website (CRD42022295587).

### Eligibility criteria

All original studies reporting the immunological response of COVID-19 vaccination in patients administered with HSCT or CAR T-cell therapy were considered for inclusion. There were no restrictions regarding language, country, and patient demographic information of the included studies due to the small volume of studies on this topic.

### Search strategy

**Fig. 1:**
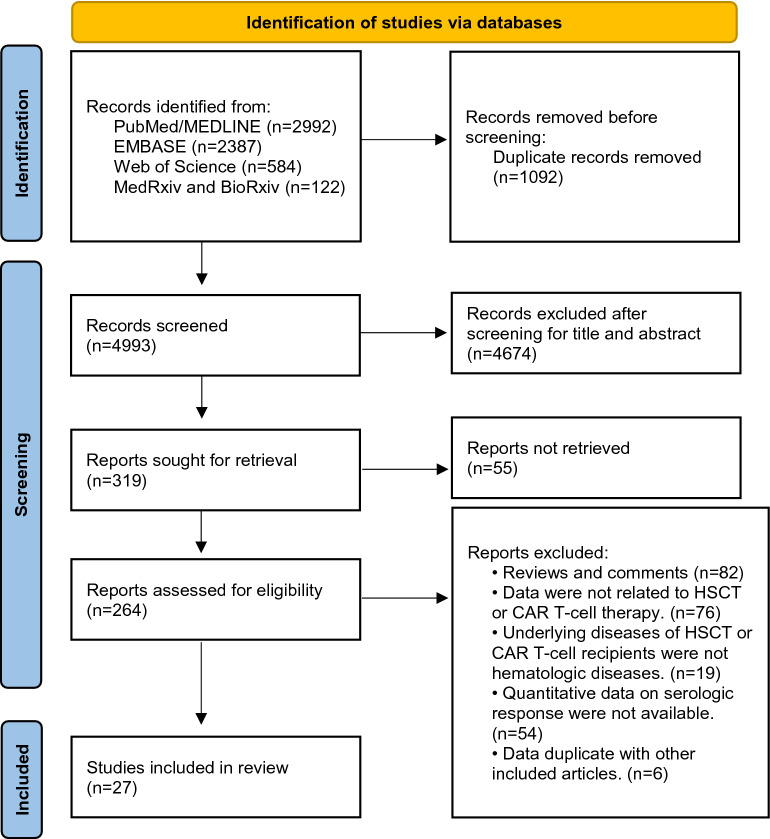
PRISMA flow diagram summarizing the process for study identification. 4993 articles were identified through the literature search. 4966 were excluded after the screening. 27 articles met eligibility criteria and were included for the analysis. HSCT: hematopoietic stem cell transplantation; CAR T-cell therapy: chimeric antigen receptor T-cell therapy

We searched in MEDLINE, EMBASE and Web of Science for peer-reviewed articles published until July 14, 2022, with mainly the following terms: COVID-19, SARS-CoV-2, vaccination, immunization, CAR T and HSCT. A similar search was performed for preprint articles in MedRvix and BioRvix. The search flowchart is presented in Fig. 1 and detailed search strategies are listed in Additional file 1: Table S2.

### Study selection and data extraction

Two reviewers (CG and GL) independently conducted literature search for potentially eligible studies, and differences in opinion were resolved by consensus among the authors. Two authors (CG and KD) independently screened the titles and abstracts to exclude studies that did not meet the inclusion criteria and resolved differences by consensus among a third author (GL). Criteria for inclusion included: (1) patients of hematologic disorders receiving HSCT or CAR T-cell therapies (For studies that did not report underlying diseases of patients, authors had to be affiliated with hematologic related institutions); (2) immunological response and/or safety outcomes were assessed post COVID-19 vaccination; and (3) results reported from original studies, other than secondary analyses. Exclusion criteria included: (1) reviews and comments; (2) the primary population in the articles were not patients receiving HSCT or CAR T-cell therapy; (3) underlying diseases were not hematologic diseases; (4) quantitative data were not available or duplicated with other studies. All key data from each included study were independently extracted by CG, KD and ML according to a pre-determined proforma and validated by GL. Data on study characteristics were extracted, including the first author’s name, study location, year of publication, sample size, underlying diseases or conditions, types of therapies, age and gender of patients, type and dose of vaccines, time to vaccination post-HSCT or CAR T-cell therapy, outcome of immunological response, safety evaluation and possible impact factors of seropositivity. Types of therapies were divided into three categories, including allo-HSCT, auto-HSCT and CAR T-cell.

### Outcomes

The primary outcome of this meta-analysis was seropositivity rate after COVID-19 vaccination, defined as the proportion of participants with positive serologic anti-SARS-CoV-2 antibodies. Quantitative IgG antibodies against SARS-CoV-2 spike protein (either RBD or S1) were tested to measure seropositivity in all included studies. The seropositivity was determined with reference to the criteria stated in each study, which varied across studies due to the differences in methodology. The secondary outcome involved adverse events or reactions after COVID-19 vaccination in HSCT or CAR T-cell recipients.

### Statistical analysis

This meta-analysis was performed with the Comprehensive Meta-Analysis Software (version 3). Random effects models were used to pool the rate of serologic response to COVID-19 vaccination among allo-HSCT, auto-HSCT, CAR T-cell therapy patients and odds ratio comparing with healthy controls. Q, I^2^ and P values were used to assess heterogeneity between studies. I^2^ value of < 25% was considered for low heterogeneity and > 75% for high heterogeneity, and p value < 0.05 for significance. Egger’s and Begg’s tests were performed to assess publication bias. Sensitivity analysis was performed by one study-removed analyses to assess the stability of the study.

### Risk of bias assessment

Given the included studies were all non-randomized observational studies, the risk of bias assessment was carried out using ROBINS-I (risk of bias in non-randomized studies of interventions) tool as previously described [22]. Briefly, studies were assessed the certainty of evidence from seven domains, including confounding, selection of participants into the study, classification of interventions, deviations from intended interventions, missing data, measurement of outcomes and selection of the reported results. Two reviewers (CG and ML) assessed each study independently, and all discrepancies were resolved by the involvement of a third reviewer (KD) in the assessment and discussion.

## Results

### Study characteristics

We identified 4993 articles through the literature search and excluded 4966 after screening. 27 articles met eligibility criteria and were included in this meta-analysis. The detailed process of literature screening is shown in Fig. 1. Among the 27 included studies, 14 were peer-reviewed full-text articles [23–36], 12 were letters or correspondences [37–48], and one was preprint [49]. In terms of vaccine types, all 27 studies involved patients administered with mRNA vaccines (BNT162b2 from Pfizer-BioNTech and mRNA-1273 from Moderna), of which five studies also contained patients with adenoviral vector vaccines (Ad26.COV2.S from Johnson & Johnson and ChAdOx1 from AstraZeneca) [24, 25, 34, 42, 47], as detailed in Table 1. A total of 2506 allo-HSCT recipients, 286 auto-HSCT, 107 CAR T-cell recipients, and 683 healthy participants with serologic data were included in this meta-analysis. All patients with reported medical history had reported a prior diagnosis of hematologic malignancies, including leukemia, lymphoma, myeloma, and myelodysplastic syndrome. Majority of the included studies did not report the number of patients with specific underlying diseases.

**Table 1:** Characteristics of the included studies

Study	Country/Year	Number of doses	Total number of patients (Number of patients with serologic response data)	Number of control group (Number of controls with serologic response data)	Underlying disease	Age	Gender (%female)	Type of vaccine received	Time to serologic response evaluation post vaccination	Antibody detection method; criteria for determining seropositivity (if avaliable)
Agnieszka Matkowska-Kocjan [23]	Poland/2021	2	65 (57) Allo-HSCT	No	ALL, CML, AML, AA, MDS, HL, others	21 (18–31)	40.0%	BNT162b2	14–21 days	Anti-SARS-CoV-2 QuantiVac ELISA IgG test (Euroimmun/PerkinElmer subsidiary, Waltham, MA, USA); ≥ 35.2 BAU/mL
Alexis Maillard [45]	France/2022	2	687 (687) Allo-HSCT	No	myeloid malignancies, lymphoid malignancies, nonmalignant disease	59 (46–66)	41.0%	BNT162b2; mRNA-1273	33 days (27–52)	1. Abbott SARS-CoV-2 IgG II Quant-test (Abbott S IgG); ≥ 50AU/ml 2. Roche Elecsys anti-SARS-CoV-2 S (Roche S tAb); ≥ 0.8U/ml
		3	181 (181) Allo-HSCT			60.5 (49.5–66.9)	39.4%		Not reported	3. DiaSorin Liaison SARS-CoV-2 TrimericS IgG (DiaSorin TriS IgG) ≥ 13AU/ml 4. Siemens SARS-CoV-2 IgG (Siemens sCOVG); ≥ 1.0 U/ml 5. Wantai SARS-CoV-2 IgG ELISA (Wantai S IgG) ≥ 0.75 AU/ml
Amandine Le Bourgeois [46]	France/2021	2	117 (117) Allo-HSCT	No	AML, MDS, MF, MDS/MF, CML, BPDCN, NHL, HL, ALL, MM, NNAA, porphyria	57 (20–75)	40.0%	BNT162b2	35 (18–77) days	Elecsys anti–SARS-CoV-2-S (Roche Elecsys, Rotkreuz, Switzerland); ≥ 0.8 U/mL
Amandine Le Bourgeois-2 [44]	France/2021	3	80 (80) Allo-HSCT	25 (25) Healthy controls	myeloid, lymphoid, others	57 (20–75)	43.8%	BNT162b2	94.5 (55–220) days	Elecsys anti–SARS-CoV-2-S (Roche Elecsys, Rotkreuz, Switzerland); ≥ 0.8 U/mL
Anne-Claire Mamez [43]	Switzerland/2021	2	63 (63) Allo-HSCT	No	Acute leukemia, MDS/MPS, lymphoid diseases, hemoglobinopathie	54 (18–78)	38.0%	BNT162b2; mRNA-1273	38 (13–98) days	Semi-quantitative Elecsys ^®^ Anti-SARSCoV-2 immunoassay (Roche); ≥ 0.8 U/mL
Binod Dhakal [47]	US/2021	1, 2	71 (71) Allo-HSCT; 45 (45) Auto-HSCT; 14 (14) CAR T	No	lymphoma, myeloma, some were not reported	25–77	not reported	BNT162b2; mRNA-1273; Ad26.COV2.S	≥ 2 weeks	Enzyme immunoassay testing antibodies to the S1 domain of the SARS-CoV-2 spike protein (EUROIMMUN)
Caroline Pabst [24]	Germany/2022	1, 2	167 (167) Allo-HSCT	134 (134) Healthy controls	AML, MDS, MPN, AA, ALL, MM, lymphoma	60 (19–79)	38.9%	BNT162b2; mRNA-1273; ChAdOx1	Not reported	Surrogate virus neutralization test (Medac, Wedel, Germany)
José Luis Piñana[25]	Spain/2021	2	311 (311) Allo-HSCT 86 (86) Auto-HSCT	No	AML, MDS, NHL, MM, CLL, HD, MPN, ALL, others	Allo: 56.7 (18–80) Auto: 64.6 (19–78)	Allo: 40.0% Auto: 43.0%	mRNA-1273; BNT162b2; ChAdOx1; Ad26.COV2.S	3–6 weeks	1. Abbott Architect SARS-CoV-2 IgG Quant II chemiluminescent microparticle immunoassay (Abbott, Sligo, Ireland) 2. Liaison SARS-CoV-2 S1/S2 IgG chemiluminescent assay (DiaSorin S.p.A., Saluggia, Italy) 3. Euroimmun SARS-CoV-2 IgG ELISA (Euroimmun, Lübeck, Germany) 4. MAGLUMI 2019-nCoV IgG chemiluminescent assay (SNIBE—Shenzhen New Industries Biomedical Engineering Co., Ltd., Shenzhen, China) 5. COVID-19 ELISA IgG (Vircell Spain S.L.U., Granada, Spain)
Kalpana Parvathaneni [41]	US/2021	2	12 (12) CAR T	8 (8) Healthy controls	B-ALL, DLBCL, NHL, MCL, CLL	53 (16–74)	25.0%	BNT162b2; mRNA-1273	Up to 28 days	Not reported
Katie Healy [35]	Sweden/2021	2	74 (69) Allo-HSCT/CAR T	82 (82) Healthy controls	Not reported	60 (51–67)	45.0%	BNT162b2	14 days	Elecsys® Anti-SARS-CoV-2 S assay (Roche Diagnostics); ≥ 0.80 U/mL
Lorenzo Canti [26]	Belgium/2021	1	40 (37) Allo-HSCT	40 (40) Healthy controls	Not reported	60 (26–76)	52.5%	BNT162b2	21 days	WANTAI SARS-Cov-2 Ab ELISA (Beijing Wantai Biological Pharmacy Enterprise, Beijing, China)
		2	40 (37) Allo-HSCT	40 (40) Healthy controls					28 days	
Lorenzo Canti-2 [39]	Belgium/2022	3	38 (38) Allo-HSCT	No	Not reported	60 (26–76)	50.0%	BNT162b2	28 days	WANTAI SARS-Cov-2 Ab ELISA (Beijing Wantai Biological Pharmacy Enterprise, Beijing, China)
Martina Chiarucci [27]	Italy/2021	2	12 (12) Allo-HSCT; 38 (38) Auto-HSCT	45 (0) Healthy controls	Auto-HSCT: MM, NHL, HL; Allo-HSCT: AML, ALL, MDS	61 (21–72)	44.0%	BNT162b2	30 days	Anti-SARSCoV-2 IgG CLIA (LIAISON ® SARS-CoV-2 TrimericS IgG assay, Diasorin, Saluggia, Italy)
Marika Watanabe [28]	Japan/2022	2	25 (25) Allo-HSCT	19 (19) Healthy controls	AML, ALL, malignant lymphoma, others	55 (23–71)	44.0%	BNT162b2	14 days (+ / − 7 days)	QuaResearch COVID-19 Human IgM IgG ELISA kit (Spike Protein-S1) (Cellspect, Inc., RCOEL961S1, Iwate, Japan); > 0.26 (optical density value)
Maciej Majcherek [29]	Poland/2022	2	64 (63) Allo-HSCT; 29 (26) Auto-HSCT;	No	AML, MM, NHL, HL, MDS, ALL	Allo-HSCT: 52 (20–68); Auto-HSCT: 58 (26–69)	Allo-HSCT: 45.0%; Auto-HSCT: 48.0%	BNT162b2	2–4 weeks	Chemiluminescent microparticle immunoassay (CMIA) “Alinity I” (Abbott Diagnostics)
Monika Lindemann [30]	Germany/2021	2	117 (117) Allo-HSCT	35 (35) Healthy controls	Acute leukemia, MDS, MPN, lymphoma, others	59 (21–77)	52.1%	BNT162b2; mRNA-1273; ChAdOx1	31 (11–137) days	CE marked Anti-SARS-CoV-2 IgG semi-quantitative ELISA (Euroimmun, Lübeck, Germany);
Muhammad Bilal Abid [48]	US/2022	3	26 (26) Allo-HSCT; 30 (30) Auto-HSCT; 10 (10) CAR T	No	Lymphoma, myeloma	31–81	32.0%	BNT162b2; mRNA-1273;	At least 14 days	AdviseDx SARS-CoV-2 IgG II assay; > 50.0 AU/mL
Noga Shem-Tov [31]	Israel/2021	2	176 (152) Allo-HSCT	272 (272) Healthy controls	AML, MDS, MPD, ALL, NHL, HL, CLL, AA	58.4 ± 14.0 (mean ± SD)	36.8%	BNT162b2	2–4 weeks	Mount Sinai Hospital Clinical Laboratory SARS-CoV-2 IgG Antibody Test;
Patrice Chevallier [32]	France/2021	1	112 (112) Allo-HSCT	26 (26) Healthy controls	AML, MDS, MF, MDS/MF, CML, BPDCN, NHL, HL, ALL, MM, AA, porphyria	57 (20–75)	40.2%	BNT162b2	16–35 days	Roche Elecsys; ≥ 0.8 U/ml
Peter Bergman [49]	Sweden/2021	2	87 (70) Allo-HSCT; 3 (2) CAR T	90 (78) Healthy controls	not reported	74% < 65	47.0%	BNT162b2	14 days	Elecsys ® AntiSARS-CoV-2 S (Roche Diagnostics); ≥ 0.8 U/ml
Rabah Redjoul [37]	France/2021	2	88 (88) Allo-HSCT	No	Myeloid malignancy, lymphoid malignancy and nonmalignant	26–77	46.6%	BNT162b2	28 (IQR 26–31) days	IgG II Quant Assay (Abbot Laboratories, Wiesbaden, Germany);
Ron Ram [36]	Israel/2021	2	66 (57) Allo-HSCT; 14 (14) CAR T	No	AML, MDS, ALL, DLBCL, other lymphoma, MPN, others	65 (23–83)	45.0%	BNT162b2	7–14 days	Elecsys Anti-SARS-CoV-2 S assay on the Cobas e411 (Roche Diagnostics, Basel, Switzerland); ≥ 0.80 U/mL
Roni Tamari [33]	US/2021	1	149 (149) Allo-HSCT; 61 (61) Auto-HSCT; 7 (7) CAR T	69 (54) Healthy controls	Acute leukemia, MDS/MPN, chronic leukemia, MM and amyloid, lymphoma, AA, SA, BPDCN	66.4 (25.8–84.1)	40.6%	BNT162b2; mRNA-1273	3 months	AdviseDx SARS-CoV-2 IgG II assay; > 50.0 AU/mL
Sandra Easdale [34]	UK/2021	1	55 (55) Allo-HSCT	No	ALL, AML, AA, MDS, NHL, HL, MF	50 (18–73)	38.2%	BNT162b2; ChAdOx1	14–84 days	Ortho Clinical Diagnostic Anti-SARS-CoV-2 IgG antibody methods (Ortho Clinical Diagnostics, USA);
Saurabh Dahiya [40]	US/2022	2	14 (14) CAR T	4 (4) Healthy controls	LBCL, MCL, FL	50.5 (24–87)	33.0%	BNT162b2; mRNA-1273	4 weeks	
		3	6 (6) CAR T	No		Not reported	Not reported		~ 1 month	
Thomas A. Fox [42]	UK/2021	2	11 (11) CAR T	No	B-ALL, NHL, CLL, WM	Not reported	Not reported	BNT162b2; ChAdOx1	1 month	Quantitative double-antigen sandwich immunoassays (Roche, Basel, Switzerland); > 0.4 µ/ml
Thomas Gastinne [38]	France/2021	1, 2	23 (23) CAR T	25 (25) Healthy controls	Lymphoma, ALL	62 (21–79)	39.1%	BNT162b2	29 (16–32) days after 1st dose and 52 (21–99) days after 2nd dose	Several serological techniques but mainly the Roche Elecsys assay

Age is expressed as median (range) or median ± quartile

*AA* aplastic anemia, *AML* acute myeloid leukemia, *ALL* acute lymphoblastic leukemia, *B-ALL* B-cell acute lymphoblastic leukemia, *BPDCN* blastic plasmacytoid dendritic cell neoplasm, *CML* chronic myelomonocytic leukemia, *DLBCL* diffuse large B cell lymphoma, *NHL* non-Hodgkin lymphoma, *HL* Hodgkin lymphoma, *LBCL* large B-cell lymphoma, *MCL* mantle cell lymphoma, *FL* follicular lymphoma, *MM* multiple myeloma, *MDS* myelodysplastic syndrome, *MF* myelofibrosis, *MPN* myeloproliferative neoplasia, *MPS* Myeloproliferative syndrome, *NNAA* nonlymphoid and nonmyeloid aplastic anemia, *SA* systemic mastocytosis, *WM* Waldenstrom macroglobulinaemia

### Risk of bias assessment

According to the ROBINS-I tool, the risk of bias was rated as low in three studies, moderate in 23 studies and serious in one study (Additional file 1: Table S3). The main source of bias was confounding, which was difficult to control due to the ever-changing pandemic of COVID-19. Unknown history of SARS-CoV-2 exposure, no matching variables such as age and underlying diseases contributed to bias in confounding and selection of participants into the study. Differences in antibody detection methods and differences in the criteria for determining seropositivity were also factors that contribute to bias in measurement of outcomes.

### Serologic response after one dose of COVID-19 vaccine

**Fig. 2:**
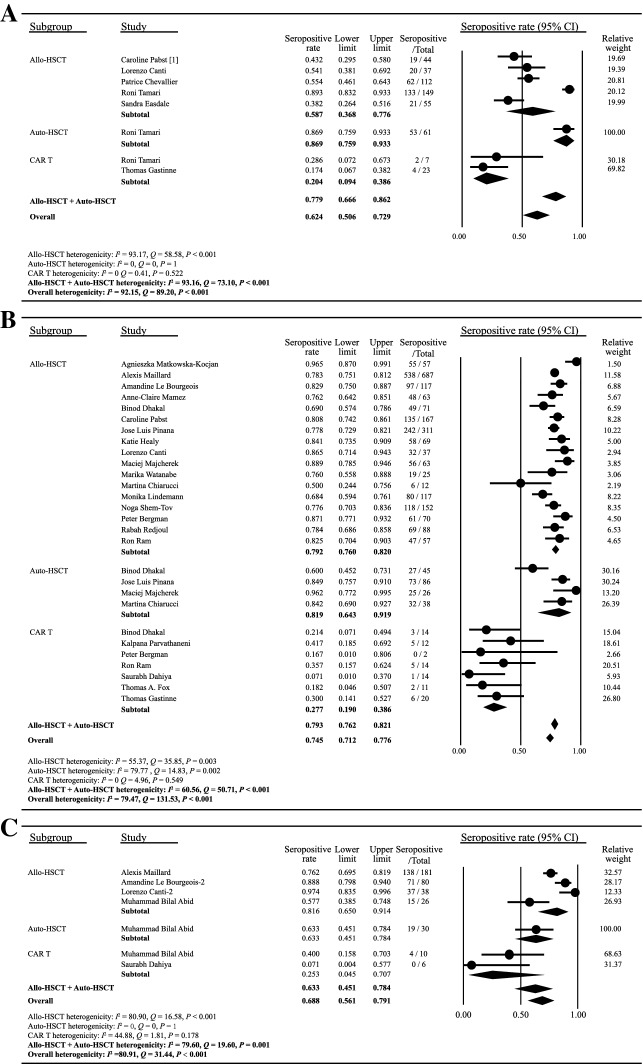
Serologic response after COVID-19 vaccination. The seroconversion rates after one dose (**A**), two doses (**B**) or three doses (**C**) of COVID-19 vaccine were plotted. The solid circles indicates the seropositivity rates, and the horizontal lines mean the 95% confidence interval (CI). The diamonds indicate the pooled estimate, and the lateral tips of the diamonds mean the 95% CIs. The event rate, lower limit, upper limit and relative weight were analyzed using the random effects models. The heterogenicity of each subgroup was represented by Q, I^2^ and P values as described in Methods

In the analysis of serologic response rates to the first dose of COVID-19 vaccination, eight cohorts from six of the included studies were available for assessment. All the six studies assessed serologic response 2 weeks to 3 months after a single dose of vaccination. As shown in Fig. 2A, the pooled proportion of HSCT or CAR T-cell recipients achieving a seropositive response was 0.624 (95% [CI] 0.506–0.729, I^2^ = 92.15). The seropositive proportion for patients with prior allo-HSCT, auto-HSCT or CAR T-cell therapeutics was 0.587 (95% [CI] 0.368–0.776, I^2^ = 93.17), 0.869 (95% [CI] 0.759–0.933, I^2^ = 0), and 0.204 (95% [CI] 0.094–0.386, I^2^ = 0), respectively. The seropositivity rate of combined allo-HSCT and auto-HSCT recipients was 0.779 (95% [CI] 0.666–0.862, I^2^ = 93.16). The funnel plot symmetry examination showed no publication bias (Begg’s test P = 0.62, Egger’s test P = 0.63) (Additional file 1: Fig. S1A). Sensitivity analyses demonstrated that there were no significant changes after removing any of the studies, with overall seroconversion rates ranging from 0.488 to 0.610 (Additional file 1: Fig. S2A).

### Serologic response after two or three doses of COVID-19 vaccine

Twenty-one of the included studies analyzed serologic responses to two doses of COVID-19 vaccine in patients receiving HSCT or CAR T-cell therapy (Fig. 2B). The vast majority of studies assessed serologic response 2–12 weeks after the second dose (Table 1). The overall proportion of seropositive response was 0.745 (95% [CI] 0.712–0.776, I^2^ = 79.47). The seropositive proportion for patients who previously received allo-HSCT, auto-HSCT or CAR T-cell therapy was 0.792 (95% [CI] 0.760–0.820, I^2^ = 55.37), 0.819 (95% [CI] 0.643–0.919, I^2^ = 79.77) and 0.277 (95% [CI] 0.190–0.386, I^2^ = 0), respectively. The overall seropositivity rate of allo-HSCT and auto-HSCT recipients was 0.793 (95% [CI] 0.762–0.821, I^2^ = 60.56) after two doses of COVID-19 vaccines. No evident publication bias was observed based on the visual symmetry of funnel plot (Begg’s test P = 0.21, Egger’s test P = 0.10) (Additional file 1: Fig. S1B). Sensitivity analyses showed that there was no significant change after removing individual studies (Additional file 1: Fig. S2B), which validated the stability and reliability of the results.

As shown in Fig. 1C, only five included studies reported the serologic response after three doses of vaccine. Four studies involved HSCT recipients and two involved CAR T-cell recipients. The overall proportion of seropositive response was 0.688 (95% [CI] 0.561–0.791, I^2^ = 80.91). Publication bias was assessed using funnel plot (Begg’s test P = 0.10, Egger’s test P = 0.56) (Additional file 1: Fig. S1C). Sensitivity analyses excluding any of the studies showed similar estimates of seropositivity (Additional file 1: Fig. S2C).

### Serologic response compared with healthy controls

**Fig. 3:**
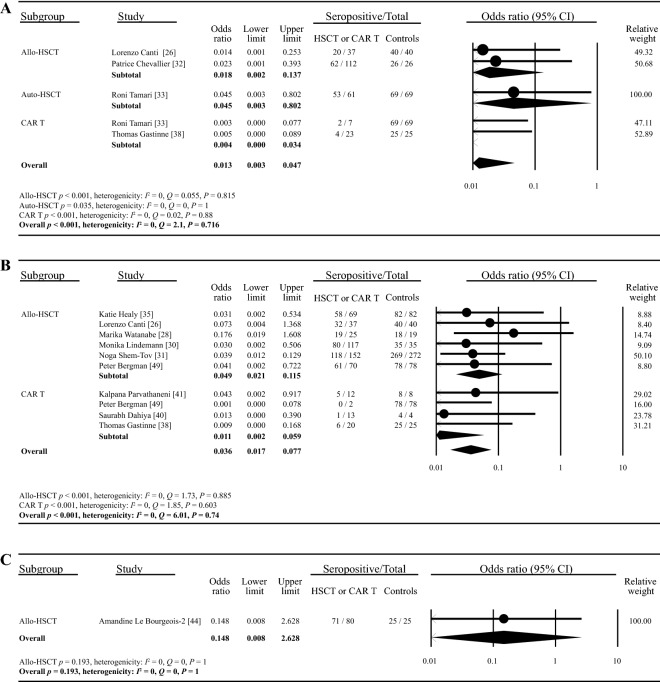
Comparison of seropositive rates between patients receiving HSCT or CAR T-cell therapy and healthy controls. The comparison of seropositive rates between HSCT or CAR T-cell recipients and healthy controls after one dose (**A**), two doses (**B**) or three doses (**C**) of COVID-19 vaccine were plotted. The solid circles indicates the odds ratio, and the horizontal lines mean the 95% CIs. The diamonds indicate the pooled estimate, and the lateral tips of the diamonds mean the 95% CIs. The event rate, lower limit, upper limit and relative weight were analyzed using the random effects models. The heterogenicity of each subgroup was represented by Q, I^2^ and P values as described in Methods

In 13 of the included studies involving healthy controls (four for one dose, ten for two doses and one for three doses), the serologic response rates of healthy people were all nearly 100%. The overall seropositive rate of HSCT and CAR T-cell recipients was significantly lower compared with that of healthy controls after one dose of vaccine (OR: 0.013, 95% [CI] 0.003–0.047, p < 0.001, I^2^ = 0) (Fig. 3A). Similar patterns were observed in the pooled analyses of serologic response after two (OR: 0.036, 95% [CI] 0.017–0.077, p < 0.001, I^2^ = 0) (Fig. 3B) or three (OR: 0.148, 95% [CI] 0.008–2.628, p = 0.193, I^2^ = 0) (Fig. 3C) doses of vaccines. The funnel plot showed no obvious publication bias for the first two settings (one dose: Begg’s test P = 0.14, Egger’s test P = 0.08; two doses: Begg’s test P = 0.09, Egger’s test P = 0.26) (Additional file 1: Fig. S1D, E). Sensitivity analysis showed that the odds ratio was not obviously altered by deselecting any studies (Additional file 1: Fig. 3A, B).

### Impact factors on seroconversion in HSCT or CAR T-cell recipients

**Fig. 4:**
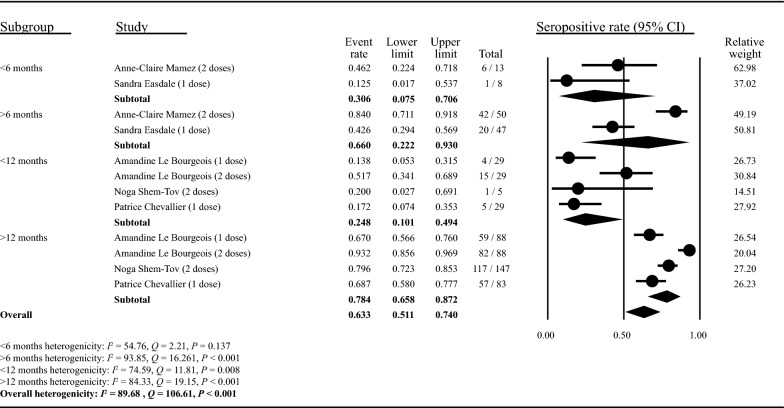
Effect of time interval between therapy to vaccination on seroconversion rate. The studies were categorized into different subgroups based on time interval between HSCT and the vaccination, referring to the classification of the time interval in the original studies. The studies included in this figure contained individuals with one or two doses vaccines and were annotated in the figure. Two studies set 6 months as the cut-off (< 6 months and > 6 months) while three studies set 12 months as the cut-off (< 12 months and > 12 months). The event rate, lower limit, upper limit and relative weight were analyzed using the random effects models. The heterogenicity of each subgroup was represented by Q, I^2^ and P values as described in Methods

Due to insufficient quantitative information, meta-regression could not be performed to assess impact factors of seroconversion rate. We therefore systematically reviewed each factor separately based on available data, with most of the conclusion on impact factors of seroconversion being derived from articles that included the HSCT recipients and few articles examined the impact factors of seroconversion in CAR T-cell recipients. (Detailed in Additional file 1: Table S5 and Fig. S4). Firstly, time interval from HSCT or CAR T to vaccination was shown to be an impactor factors on seropositivity rate in 14 included studies [25, 26, 28–33, 36, 37, 43, 45, 46, 49], and no significant correlation in 6 included studies [23, 24, 34, 38, 47, 48]. Subgroup analysis also showed a possibly positive effect of time interval on seropositivity rate (Fig. 4). More than 6-month time interval indicated a better seroconversion than that less than 6 months (0.660 [0.222–0.930] vs 0.306 [0.075–0.706]), and more than 12 months also indicated a better seroconversion (0.784 [0.658–0.872] vs 0.248 [0.101–0.494]). Furthermore, the use of immunosuppressants pre-HSCT or post-HSCT was found to have a negative impact on antibody response in 13 studies [24, 25, 27–29, 31–34, 37, 43, 45, 46], but found no evident correlation in other 4 studies [23, 36, 47, 48]. The number of lymphocytes was also demonstrated to be correlated with seroconversion in 14 included studies [24–29, 32, 33, 36, 37, 41, 43, 45, 46], especially CD4^+^ T cells, CD19^+^ T cells and B cells, and no significant correlation in 5 studies [23, 31, 34, 47, 48]. The presence of chronic graft-versus-host disease (cGVHD) could also be a contributing factor for seroconversion in only 4 included studies [26, 29, 31, 49].

### Safety evaluation after COVID-19 vaccination in HSCT or CAR T-cell recipients

14 of the included studies reported adverse events or reactions after COVID-19 vaccination in patients receiving HSCT or CAR T-cell therapy (Additional file 1: Table S4). Common (incidence rates higher than 10%) local reactions at injection site included pain, swelling and redness, and common systemic adverse reactions included fever, chills, fatigue, myalgias and arthralgias, which were reported in at least four studies [23, 28, 32, 38]. Most adverse reactions were mild (grade 1 or 2) and could be resolved within a few days [23, 25, 28, 32, 38, 46]. Two studies found the presence of vaccine-related hematologic adverse reactions, including cytopenia exacerbation (12.5%) and GVHD exacerbation (4.5%), and the reactions were also resolved quickly [24, 36]. No grade 3 or 4 adverse event was reported. Overall, the COVID-19 mRNA vaccination was relatively safe for HSCT and CAR T-cell recipients.

## Discussion

Patients with hematologic diseases receiving HSCT or CAR T-cell therapy are at an increased risk of morbidity and mortality associated with COVID-19 [18, 50]. Whether and when these patients should receive COVID-19 vaccines has become a critical issue. To our knowledge, the present study is the first systematic review and meta-analysis to examine the serologic response and safety of COVID-19 vaccines in patients who have undergone HSCT or CAR T-cell therapy. Our findings suggested that these patients exhibited an impaired serologic response to COVID-19 vaccination, in reference to the approaching 100% response of the healthy controls included in the studies. The seroconversion rate might increase after repeated inoculations. Our analysis found a seemingly increasing trend in seropositivity with repeated shots, ranging from 0.624 (95% [CI] 0.506–0.729) after one dose to 0.745 (95% [CI] 0.712–0.776) after two doses and 0.688 (95% [CI] 0.561–0.791) after three doses. Overall, our findings suggest that HSCT or CAR T-cell recipients are encouraged to receive a full course of COVID-19 vaccination and booster shots. Non-pharmaceutical interventions like wearing masks and maintaining social distance have also been identified as effective measures to interrupt virus transmission [51], which are even more important for HSCT or CAR T-cell recipients due to the weakened vaccine protection.

When looking at different types of therapies, we demonstrated that patients receiving CAR T-cell garnered a substantially lower serologic response compared to those receiving HSCT (one dose: 0.204 [0.094–0.386] vs 0.779 [0.666–0.862]); two doses: 0.277 [0.190–0.386] vs 0.792 [0.761–0.819]), suggesting that CAR T-cell therapy might lead to a poorer response to COVID-19 vaccination. CAR T-cell recipients usually have a background of B-cell related malignancies, such as refractory large B-cell lymphoma and B-cell lymphocytic leukemia [52, 53]. These patients are prone to exhibit sustained low or even nondetectable circulating B cells after CAR T-cell cell infusion [54], which might explain the particularly low seroconversion rate. Indeed, impaired seroprotecting for vaccine-preventable infections was reported in CAR T-cell recipients, especially in BCMA-targeted CAR T-cell cell recipients [55]. Another possible interpretation lies in the patients included in this meta-analysis had a variety of hematologic disorders with varying disease states. Previous studies have demonstrated that patients with hematologic disorders react to COVID-19 vaccination in a variable manner, depending on type and activity level of the disorder [56–58]. However, the information of specific diagnosis for individual patients were lacking for most studies. In addition, HSCT and CAR T-cell recipients may receive different immunosuppressive treatments and conditioning regimens which might also introduce bias in the assessment of seropositive rate.

Possible factors affecting seropositive rate were analyzed in this study, including therapy-vaccination time interval, immunosuppressive treatment and immune cell counts. European and US transplant guidelines suggested that COVID-19 vaccination for HSCT or CAR T-cell recipients can be initiated 3 months after treatment, which is rational in regions with severe outbreaks to minimize infection chance. The consensus guidelines recommend receiving vaccines for other pathogens 6 months after HSCT to obtain a better seroconversion [59, 60], and similar results were notified in this systematic review. In summary, the adoption of an appropriate interval (more than 6 months) between vaccination and the HSCT or CAR T-cell therapy may provide a higher serologic response rate. Consistent with our findings, previous studies have demonstrated that the prior use of immunosuppressants and low number of lymphocytes were associated with attenuated serologic response to COVID-19 vaccination in patients with immune-mediated inflammatory diseases addressed in a meta-analysis [61]. However, given a lack of quantitative information, meta-regression of impact factors on seroconversion was not conducted and hard to draw a solid conclusion. and larger prospective studies are needed to confirm the association.

Furthermore, vaccination with COVID-19 vaccines was generally tolerated in HSCT or CAR T-cell recipients, with the majority of adverse reactions being mild and transient, comparable to those in the general population [62]. Nevertheless, considering the limited patient pool included, certain important but possibly rare safety alarms might not be captured in these retrospective studies. Therefore, active monitoring is required after vaccination and cautions must be paid on some hematologic events given the underlying hematologic disorders of HSCT or CAR T-cell recipients.

This study has some limitations. First, the included studies were limited in patient number and showed high heterogeneity. Reasons for high statistical heterogeneity in this meta-analysis included differences in baseline characteristics of included patients, such as age, underlying diseases and serologic testing method. Multiple COVID-19 antibody testing kits were applied in different studies, and the criteria for defining seroconversion positivity varied among institutions. Second, meta-regression could not be conducted due to insufficient information. Another limitation came from lack of evidence for long-term effectiveness and safety of the vaccines. Due to the urgency of vaccine development, most of the included studies were only followed up to 3 months post-vaccination. Longer follow-up is needed to determine whether the level of neutralizing antibodies can be maintained over time and whether there are late-onset adverse reactions after vaccination. In addition, the response to COVID-19 vaccination was only analyzed by positive humoral response, while the cellular immunity was not presented. An additional limitation arises from that the vast majority of vaccine types included in the studies were mRNA vaccines, or serological data for different vaccines were not differentiated in some individual studies. The data on other types (inactivated vaccine, viral vector vaccine and protein subunit vaccine) of vaccines were lacking. Finally, only seroconversion rates were discussed in this study, and data on vaccine efficacy in real-world were lacking, although vaccine efficacy was thought to be correlated with seroconversion [63].

## Conclusion

Our meta-analysis reported an impaired humoral response to COVID-19 vaccines in HSCT recipients, and the response might be even lower in CAR T-cell recipients. Better seropositivity rate might be achieved when the interval between therapy and vaccination exceeds 6 months. Regularly repeated COVID-19 vaccination at appropriate intervals after HSCT or CAR T-cell therapy is probably beneficial for these patients. Further studies assessing the responses and protection to the fourth dose and other types of vaccines are warranted.

## Supplementary Information

**Additional file 1: **Table S1 PRISMA checklist. **Table S2.** Search strategy. **Table S3.** Risk of bias assessment. **Table S4.** Safety evaluation after COVID-19 vaccination in patients receiving HSCT or CAR T therapy. **Table S5.** Impact factors of seroconversion rate in HSCT or CAR T recipients. **Figure S1.** Funnel plots. Funnel plot analysis of studies in the meta-analysis. (A) Serologic response after one dose of vaccine. (B) Serologic response after two doses of vaccine. (C) Serologic response after three doses of vaccine. (D) Comparison of seropositive rate in recipients and healthy controls after 1 dose of vaccine. (E) Comparison of seropositive rate in recipients and healthy controls after 2 doses of vaccine. **Figure S2.** Sensitivity analysis excluding one subgroup within a study at a time for “serologic response after COVID-19 vaccination”. (A) Sensitivity analysis for serologic response after one dose of vaccine. (B) Sensitivity analysis for serologic response after two doses of vaccine. (C) Sensitivity analysis for serologic response after three doses of vaccine. The size of the solid circles denotes the mean difference, and the horizontal lines represent the 95% CIs. The diamond denotes the pooled estimate, and the lateral tips of the diamond indicate the 95% CIs. **Figure S3.** Sensitivity analysis excluding one subgroup within a study at a time for “comparison of patients receiving HSCT or CAR T therapy with healthy controls”. (A) Sensitivity analysis for comparison of serologic response after one dose of vaccine. (B) Sensitivity analysis for comparison of serologic response after two doses of vaccine. The size of the solid circles denotes the mean difference, and the horizontal lines represent the 95% CIs. The diamond denotes the pooled estimate, and the lateral tips of the diamond indicate the 95% CIs. **Figure S4.** Impact factors of seroconversion in HSCT or CAR T-cell recipients. The impact factors of seroconversion in HSCT or CAR T-cell recipients were summarized in four aspacts as annotated in the figure. They were divided into three categories (significant correlation, no significant correlation, not reported) based on the correlation with seroconversion.

## Data Availability

Not applicable.

## References

[1] World Health Organization. 2022. https://covid19.who.int/. Accessed 15 July 2022.

[2] Dagan N, Barda N, Kepten E, Miron O, Perchik S, Katz MA (2021). BNT162b2 mRNA Covid-19 vaccine in a nationwide mass vaccination setting. N Engl J Med.

[3] CDC Expands Eligibility for COVID-19 Booster Shots to All Adults. https://www.cdc.gov/media/releases/2021/s1119-booster-shots.html. Accessed 01 Apr 2022.

[4] Lopez Bernal J, Andrews N, Gower C, Robertson C, Stowe J, Tessier E (2021). Effectiveness of the Pfizer-BioNTech and Oxford-AstraZeneca vaccines on covid-19 related symptoms, hospital admissions, and mortality in older adults in England: test negative case-control study. BMJ (Clinical research ed).

[5] Haas EJ, Angulo FJ, McLaughlin JM, Anis E, Singer SR, Khan F (2021). Impact and effectiveness of mRNA BNT162b2 vaccine against SARS-CoV-2 infections and COVID-19 cases, hospitalisations, and deaths following a nationwide vaccination campaign in Israel: an observational study using national surveillance data. Lancet (London, England).

[6] Voysey M, Costa Clemens SA, Madhi SA, Weckx LY, Folegatti PM, Aley PK (2021). Single-dose administration and the influence of the timing of the booster dose on immunogenicity and efficacy of ChAdOx1 nCoV-19 (AZD1222) vaccine: a pooled analysis of four randomised trials. Lancet (London, England).

[7] LABEL: PFIZER-BIONTECH COVID-19 VACCINE- bnt162b2 injection, suspension. https://www.dailymed.nlm.nih.gov/dailymed/drugInfo.cfm?setid=908ecbe7-2f1b-42dd-94bf-f917ec3c5af8 Accessed 01 Apr 2022.

[8] Holstein SA, Lunning MA (2020). CAR T-cell therapy in hematologic malignancies: a voyage in progress. Clin Pharmacol Ther.

[9] Larson RC, Maus MV (2021). Recent advances and discoveries in the mechanisms and functions of CAR T cells. Nat Rev Cancer.

[10] Eaves CJ (2015). Hematopoietic stem cells: concepts, definitions, and the new reality. Blood.

[11] Sharrack B, Saccardi R, Alexander T, Badoglio M, Burman J, Farge D (2020). Autologous haematopoietic stem cell transplantation and other cellular therapy in multiple sclerosis and immune-mediated neurological diseases: updated guidelines and recommendations from the EBMT Autoimmune Diseases Working Party (ADWP) and the Joint Accreditation Committee of EBMT and ISCT (JACIE). Bone Marrow Transplant.

[12] Barriga F, Ramírez P, Wietstruck A, Rojas N (2012). Hematopoietic stem cell transplantation: clinical use and perspectives. Biol Res.

[13] Gyurkocza B, Lazarus HM, Giralt S (2017). Allogeneic hematopoietic cell transplantation in patients with AML not achieving remission: potentially curative therapy. Bone Marrow Transplant.

[14] Brentjens RJ, Davila ML, Riviere I, Park J, Wang X, Cowell LG (2013). CD19-targeted T cells rapidly induce molecular remissions in adults with chemotherapy-refractory acute lymphoblastic leukemia. Sci Transl Med.

[15] Raje N, Berdeja J, Lin Y, Siegel D, Jagannath S, Madduri D (2019). Anti-BCMA CAR T-cell therapy bb2121 in relapsed or refractory multiple myeloma. N Engl J Med.

[16] Bupha-Intr O, Haeusler G, Chee L, Thursky K, Slavin M, Teh B (2021). CAR-T cell therapy and infection: a review. Expert Rev Anti Infect Ther.

[17] Peinemann F, Smith LA, Kromp M, Bartel C, Kröger N, Kulig M (2013). Autologous hematopoietic stem cell transplantation following high-dose chemotherapy for non-rhabdomyosarcoma soft tissue sarcomas. Cochrane database Syst Rev.

[18] Sharma A, Bhatt NS, St Martin A, Abid MB, Bloomquist J, Chemaly RF (2021). Clinical characteristics and outcomes of COVID-19 in haematopoietic stem-cell transplantation recipients: an observational cohort study. Lancet Haematol.

[19] Spanjaart AM, Ljungman P, de La Camara R, Tridello G, Ortiz-Maldonado V, Urbano-Ispizua A (2021). Poor outcome of patients with COVID-19 after CAR T-cell therapy for B-cell malignancies: results of a multicenter study on behalf of the European Society for Blood and Marrow Transplantation (EBMT) Infectious Diseases Working Party and the European Hematology Association (EHA) Lymphoma Group. Leukemia.

[20] Borek AJ, Maitland K, McLeod M, Campbell A, Hayhoe B, Butler CC (2021). Impact of the COVID-19 pandemic on community antibiotic prescribing and stewardship: a qualitative interview study with general practitioners in England. medRxiv.

[21] EBMT: COVID-19 vaccines. Version 8. https://www.ebmt.org/covid-19-and-bmt Accessed 01 Mar 2022.

[22] Sterne JA, Hernán MA, Reeves BC, Savović J, Berkman ND, Viswanathan M (2016). ROBINS-I: a tool for assessing risk of bias in non-randomised studies of interventions. BMJ.

[23] Matkowska-Kocjan A, Owoc-Lempach J, Chruszcz J, Kuźnik E, Szenborn F, Jurczenko L (2021). The COVID-19 mRNA BNT163b2 vaccine was well tolerated and highly immunogenic in young adults in long follow-up after haematopoietic stem cell transplantation. Vaccines.

[24] Pabst C, Benning L, Liebers N, Janssen M, Caille L, Speer C (2022). Humoral responses and chronic GVHD exacerbation after COVID-19 vaccination post allogeneic stem cell transplantation. Vaccines.

[25] Piñana JL, López-Corral L, Martino R, Montoro J, Vazquez L, Pérez A (2022). SARS-CoV-2-reactive antibody detection after SARS-CoV-2 vaccination in hematopoietic stem cell transplant recipients: prospective survey from the Spanish Hematopoietic Stem Cell Transplantation and Cell Therapy Group. Am J Hematol.

[26] Canti L, Humblet-Baron S, Desombere I, Neumann J, Pannus P, Heyndrickx L (2021). Predictors of neutralizing antibody response to BNT162b2 vaccination in allogeneic hematopoietic stem cell transplant recipients. J Hematol Oncol.

[27] Chiarucci M, Paolasini S, Isidori A, Guiducci B, Loscocco F, Capalbo M (2021). Immunological response against SARS-COV-2 After BNT162b2 vaccine administration is impaired in allogeneic but not in autologous stem cell transplant recipients. Front Oncol.

[28] Watanabe M, Yakushijin K, Funakoshi Y, Ohji G, Hojo W, Sakai H (2022). The safety and immunogenicity of the BNT162b2 mRNA COVID-19 vaccine in Japanese patients after allogeneic stem cell transplantation. Vaccines.

[29] Majcherek M, Matkowska-Kocjan A, Szymczak D, Karasek M, Szeremet A, Kiraga A (2022). Two doses of BNT162b2 mRNA vaccine in patients after hematopoietic stem cell transplantation: humoral response and serological conversion predictors. Cancers.

[30] Lindemann M, Klisanin V, Thümmler L, Fisenkci N, Tsachakis-Mück N, Ditschkowski M (2021). Humoral and cellular vaccination responses against SARS-CoV-2 in hematopoietic stem cell transplant recipients. Vaccines.

[31] Shem-Tov N, Yerushalmi R, Danylesko I, Litachevsky V, Levy I, Olmer L (2022). Immunogenicity and safety of the BNT162b2 mRNA COVID-19 vaccine in haematopoietic stem cell transplantation recipients. Br J Haematol.

[32] Chevallier P, Coste-Burel M, Le Bourgeois A, Peterlin P, Garnier A, Béné MC (2021). Safety and immunogenicity of a first dose of SARS-CoV-2 mRNA vaccine in allogeneic hematopoietic stem-cells recipients. EJHaem.

[33] Tamari R, Politikos I, Knorr DA, Vardhana SA, Young JC, Marcello LT (2021). Predictors of humoral response to SARS-CoV-2 vaccination after hematopoietic cell transplantation and CAR T-cell therapy. Blood Cancer Discov.

[34] Easdale S, Shea R, Ellis L, Bazin J, Davis K, Dallas F (2021). Serologic responses following a single dose of SARS-Cov-2 vaccination in allogeneic stem cell transplantation recipients. Transplant Cell Ther.

[35] Healy K, Pin E, Chen P, Söderdahl G, Nowak P, Mielke S (2022). Salivary IgG to SARS-CoV-2 indicates seroconversion and correlates to serum neutralization in mRNA-vaccinated immunocompromised individuals. Med (New York, NY).

[36] Ram R, Hagin D, Kikozashvilli N, Freund T, Amit O, Bar-On Y (2021). Safety and immunogenicity of the BNT162b2 mRNA COVID-19 vaccine in patients after allogeneic HCT or CD19-based CART therapy-a single-center prospective cohort study. Transplant Cell Ther.

[37] Redjoul R, Le Bouter A, Beckerich F, Fourati S, Maury S (2021). Antibody response after second BNT162b2 dose in allogeneic HSCT recipients. Lancet (London, England).

[38] Gastinne T, Le Bourgeois A, Coste-Burel M, Guillaume T, Peterlin P, Garnier A (2022). Safety and antibody response after one and/or two doses of BNT162b2 Anti-SARS-CoV-2 mRNA vaccine in patients treated by CAR T cells therapy. Br J Haematol.

[39] Canti L, Ariën KK, Desombere I, Humblet-Baron S, Pannus P, Heyndrickx L (2022). Antibody response against SARS-CoV-2 Delta and Omicron variants after third-dose BNT162b2 vaccination in allo-HCT recipients. Cancer Cell.

[40] Dahiya S, Luetkens T, Lutfi F, Avila S, Iraguha T, Margiotta P (2022). Impaired immune response to COVID-19 vaccination in patients with B-cell malignancies after CD19 CAR T-cell therapy. Blood Adv.

[41] Parvathaneni K, Toress-Rodriguez K, Meng W, Knox J, Xu X, Weiskopf D (2021). Adoptive immune responses to Sars-Cov2 vaccination in CART19 treated patients. Blood.

[42] Fox TA, Kirkwood AA, Enfield L, O'Reilly M, Arulogun S, D'Sa S (2021). Low seropositivity and suboptimal neutralisation rates in patients fully vaccinated against COVID-19 with B-cell malignancies. Br J Haematol.

[43] Mamez AC, Pradier A, Giannotti F, Petitpas A, Fabra Urdiola M, Vu-Cantero D (2021). Antibody responses to SARS-CoV2 vaccination in a high proportion of allogeneic hematopoietic stem cell transplant recipients. Swiss Med Wkly.

[44] Le Bourgeois A, Coste-Burel M, Guillaume T, Peterlin P, Garnier A, Imbert BM (2022). Interest of a third dose of BNT162b2 anti-SARS-CoV-2 messenger RNA vaccine after allotransplant. Br J Haematol.

[45] Maillard A, Redjoul R, Klemencie M, Labussière Wallet H, Le Bourgeois A, D'Aveni M (2022). Antibody response after 2 and 3 doses of SARS-CoV-2 mRNA vaccine in allogeneic hematopoietic cell transplant recipients. Blood.

[46] Le Bourgeois A, Coste-Burel M, Guillaume T, Peterlin P, Garnier A, Béné MC (2021). Safety and Antibody response after 1 and 2 doses of BNT162b2 mRNA vaccine in recipients of allogeneic hematopoietic stem cell transplant. JAMA Netw Open.

[47] Dhakal B, Abedin S, Fenske T, Chhabra S, Ledeboer N, Hari P (2021). Response to SARS-CoV-2 vaccination in patients after hematopoietic cell transplantation and CAR T-cell therapy. Blood.

[48] Abid MB, Rubin M, Ledeboer N, Szabo A, Longo W, Mohan M (2022). Efficacy of a third SARS-CoV-2 mRNA vaccine dose among hematopoietic cell transplantation, CAR T cell, and BiTE recipients. Cancer Cell.

[49] Bergman P, Blennow O, Hansson L, Mielke S, Nowak P, Chen P (2021). Safety and efficacy of the mRNA BNT162b2 vaccine against SARS-CoV-2 in five groups of immunocompromised patients and healthy controls in a prospective open-label clinical trial. medRxiv.

[50] Busca A, Salmanton-García J, Corradini P, Marchesi F, Cabirta A, Di Blasi R (2021). COVID-19 and CAR-T cells: current challenges and future directions-a report from the EPICOVIDEHA survey by EHA-IDWP. Blood Adv.

[51] Talic S, Shah S, Wild H, Gasevic D, Maharaj A, Ademi Z (2021). Effectiveness of public health measures in reducing the incidence of covid-19, SARS-CoV-2 transmission, and covid-19 mortality: systematic review and meta-analysis. BMJ (Clinical research ed).

[52] Neelapu SS, Locke FL, Bartlett NL, Lekakis LJ, Miklos DB, Jacobson CA (2017). Axicabtagene ciloleucel CAR T-cell therapy in refractory large B-cell lymphoma. N Engl J Med.

[53] Maude SL, Laetsch TW, Buechner J, Rives S, Boyer M, Bittencourt H (2018). Tisagenlecleucel in children and young adults with B-cell lymphoblastic leukemia. N Engl J Med.

[54] Shah NN, Johnson BD, Schneider D, Zhu F, Szabo A, Keever-Taylor CA (2020). Bispecific anti-CD20, anti-CD19 CAR T cells for relapsed B cell malignancies: a phase 1 dose escalation and expansion trial. Nat Med.

[55] Walti CS, Krantz EM, Maalouf J, Boonyaratanakornkit J, Keane-Candib J, Joncas-Schronce L (2021). Antibodies against vaccine-preventable infections after CAR-T cell therapy for B cell malignancies. JCI Insight..

[56] Mori A, Onozawa M, Tsukamoto S, Ishio T, Yokoyama E, Izumiyama K (2022). Humoral response to mRNA-based COVID-19 vaccine in patients with myeloid malignancies. Br J Haematol.

[57] Herishanu Y, Avivi I, Aharon A, Shefer G, Levi S, Bronstein Y (2021). Efficacy of the BNT162b2 mRNA COVID-19 vaccine in patients with chronic lymphocytic leukemia. Blood.

[58] Perry C, Luttwak E, Balaban R, Shefer G, Morales MM, Aharon A (2021). Efficacy of the BNT162b2 mRNA COVID-19 vaccine in patients with B-cell non-Hodgkin lymphoma. Blood Adv.

[59] Rubin LG, Levin MJ, Ljungman P, Davies EG, Avery R, Tomblyn M (2014). 2013 IDSA clinical practice guideline for vaccination of the immunocompromised host. Clin Infect Dis.

[60] Cordonnier C, Einarsdottir S, Cesaro S, Di Blasi R, Mikulska M, Rieger C (2019). Vaccination of haemopoietic stem cell transplant recipients: guidelines of the 2017 European Conference on Infections in Leukaemia (ECIL 7). Lancet Infect Dis.

[61] Sakuraba A, Luna A, Micic D (2022). Serologic response to coronavirus disease 2019 (COVID-19) vaccination in patients with immune-mediated inflammatory diseases: a systematic review and meta-analysis. Gastroenterology.

[62] Centers for disease control, 2021. https://www.cdc.gov/vaccines/covid-19/info-by-product/pfizer/reactogenicity.html. Accessed 01 Apr 2022.

[63] Gilbert PB, Montefiori DC, McDermott AB, Fong Y, Benkeser D, Deng W (2022). Immune correlates analysis of the mRNA-1273 COVID-19 vaccine efficacy clinical trial. Science (New York, NY).

