# Supplementary material for: Serologic response and safety of COVID-19 vaccination in HSCT or CAR T-cell recipients: a systematic review and meta-analysis
Source: Exp Hematol Oncol. 2022 Aug 16;11:46. doi: 10.1186/s40164-022-00299-6 (PMC9380660; doi:10.1186/s40164-022-00299-6)
Supplement: Supplementary file 1 — Additional file 1: Table S1 PRISMA checklist. Table S2. Search strategy. Table S3. Risk of bias assessment. Table S4. Safety evaluation after COVID-19 vaccination in patients receiving HSCT or CAR T therapy. Table S5. Impact factors of seroconversion rate in HSCT or CAR T recipients. Figure S1. Funnel plots. Funnel plot analysis of studies in the meta-analysis. (A) Serologic response after one dose of vaccine. (B) Serologic response after two doses of vaccine. (C) Serologic response after three doses of vaccine. (D) Comparison of seropositive rate in recipients and healthy controls after 1 dose of vaccine. (E) Comparison of seropositive rate in recipients and healthy controls after 2 doses of vaccine. Figure S2. Sensitivity analysis excluding one subgroup within a study at a time for “serologic response after COVID-19 vaccination”. (A) Sensitivity analysis for serologic response after one dose of vaccine. (B) Sensitivity analysis for serologic response after two doses of vaccine. (C) Sensitivity analysis for serologic response after three doses of vaccine. The size of the solid circles denotes the mean difference, and the horizontal lines represent the 95% CIs. The diamond denotes the pooled estimate, and the lateral tips of the diamond indicate the 95% CIs. Figure S3. Sensitivity analysis excluding one subgroup within a study at a time for “comparison of patients receiving HSCT or CAR T therapy with healthy controls”. (A) Sensitivity analysis for comparison of serologic response after one dose of vaccine. (B) Sensitivity analysis for comparison of serologic response after two doses of vaccine. The size of the solid circles denotes the mean difference, and the horizontal lines represent the 95% CIs. The diamond denotes the pooled estimate, and the lateral tips of the diamond indicate the 95% CIs. Figure S4. Impact factors of seroconversion in HSCT or CAR T-cell recipients. The impact factors of seroconversion in HSCT or CAR T-cell recipients were summarized in four [file 40164_2022_299_MOESM1_ESM.docx]

Supplementary Table 1 PRISMA checklist

| Section and Topic | Item # | Checklist item | Location where item is reported |
| --- | --- | --- | --- |
| TITLE | | | Pages |
| Title | 1 | Identify the report as a systematic review. | 1 |
| ABSTRACT | | |  |
| Abstract | 2 | See the PRISMA 2020 for Abstracts checklist. | 2 |
| INTRODUCTION | | |  |
| Rationale | 3 | Describe the rationale for the review in the context of existing knowledge. | 3 |
| Objectives | 4 | Provide an explicit statement of the objective(s) or question(s) the review addresses. | 3 |
| METHODS | | |  |
| Eligibility criteria | 5 | Specify the inclusion and exclusion criteria for the review and how studies were grouped for the syntheses. | 4 |
| Information sources | 6 | Specify all databases, registers, websites, organisations, reference lists and other sources searched or consulted to identify studies. Specify the date when each source was last searched or consulted. | 4 |
| Search strategy | 7 | Present the full search strategies for all databases, registers and websites, including any filters and limits used. | 4 |
| Selection process | 8 | Specify the methods used to decide whether a study met the inclusion criteria of the review, including how many reviewers screened each record and each report retrieved, whether they worked independently, and if applicable, details of automation tools used in the process. | 4 |
| Data collection process | 9 | Specify the methods used to collect data from reports, including how many reviewers collected data from each report, whether they worked independently, any processes for obtaining or confirming data from study investigators, and if applicable, details of automation tools used in the process. | 4,5 |
| Data items | 10a | List and define all outcomes for which data were sought. Specify whether all results that were compatible with each outcome domain in each study were sought (e.g. for all measures, time points, analyses), and if not, the methods used to decide which results to collect. | 5 |
|  | 10b | List and define all other variables for which data were sought (e.g. participant and intervention characteristics, funding sources). Describe any assumptions made about any missing or unclear information. | 5 |
| Study risk of bias assessment | 11 | Specify the methods used to assess risk of bias in the included studies, including details of the tool(s) used, how many reviewers assessed each study and whether they worked independently, and if applicable, details of automation tools used in the process. | 5 |
| Effect measures | 12 | Specify for each outcome the effect measure(s) (e.g. risk ratio, mean difference) used in the synthesis or presentation of results. | 5 |
| Synthesis methods | 13a | Describe the processes used to decide which studies were eligible for each synthesis (e.g. tabulating the study intervention characteristics and comparing against the planned groups for each synthesis (item #5)). | 5 |
|  | 13b | Describe any methods required to prepare the data for presentation or synthesis, such as handling of missing summary statistics, or data conversions. | 5 |
|  | 13c | Describe any methods used to tabulate or visually display results of individual studies and syntheses. | 5 |
|  | 13d | Describe any methods used to synthesize results and provide a rationale for the choice(s). If meta-analysis was performed, describe the model(s), method(s) to identify the presence and extent of statistical heterogeneity, and software package(s) used. | 5 |
|  | 13e | Describe any methods used to explore possible causes of heterogeneity among study results (e.g. subgroup analysis, meta-regression). | 5 |
|  | 13f | Describe any sensitivity analyses conducted to assess robustness of the synthesized results. | 5 |
| Reporting bias assessment | 14 | Describe any methods used to assess risk of bias due to missing results in a synthesis (arising from reporting biases). | 5 |
| Certainty assessment | 15 | Describe any methods used to assess certainty (or confidence) in the body of evidence for an outcome. | 5 |
| RESULTS | | |  |
| Study selection | 16a | Describe the results of the search and selection process, from the number of records identified in the search to the number of studies included in the review, ideally using a flow diagram. | 6 |
|  | 16b | Cite studies that might appear to meet the inclusion criteria, but which were excluded, and explain why they were excluded. | 6 |
| Study characteristics | 17 | Cite each included study and present its characteristics. | 6 |
| Risk of bias in studies | 18 | Present assessments of risk of bias for each included study. | 6 |
| Results of individual studies | 19 | For all outcomes, present, for each study: (a) summary statistics for each group (where appropriate) and (b) an effect estimate and its precision (e.g. confidence/credible interval), ideally using structured tables or plots. | 7,8 |
| Results of syntheses | 20a | For each synthesis, briefly summarise the characteristics and risk of bias among contributing studies. | 7,8 |
|  | 20b | Present results of all statistical syntheses conducted. If meta-analysis was done, present for each the summary estimate and its precision (e.g. confidence/credible interval) and measures of statistical heterogeneity. If comparing groups, describe the direction of the effect. | 7,8 |
|  | 20c | Present results of all investigations of possible causes of heterogeneity among study results. | 7,8 |
|  | 20d | Present results of all sensitivity analyses conducted to assess the robustness of the synthesized results. | 7,8 |
| Reporting biases | 21 | Present assessments of risk of bias due to missing results (arising from reporting biases) for each synthesis assessed. | 7,8 |
| Certainty of evidence | 22 | Present assessments of certainty (or confidence) in the body of evidence for each outcome assessed. | 7,8 |
| DISCUSSION | | |  |
| Discussion | 23a | Provide a general interpretation of the results in the context of other evidence. | 8,9 |
|  | 23b | Discuss any limitations of the evidence included in the review. | 10 |
|  | 23c | Discuss any limitations of the review processes used. | 10 |
|  | 23d | Discuss implications of the results for practice, policy, and future research. | 10 |
| OTHER INFORMATION | | |  |
| Registration and protocol | 24a | Provide registration information for the review, including register name and registration number, or state that the review was not registered. | 1 |
|  | 24b | Indicate where the review protocol can be accessed, or state that a protocol was not prepared. | 1 |
|  | 24c | Describe and explain any amendments to information provided at registration or in the protocol. | 1 |
| Support | 25 | Describe sources of financial or non-financial support for the review, and the role of the funders or sponsors in the review. | - |
| Competing interests | 26 | Declare any competing interests of review authors. | 11 |
| Availability of data, code and other materials | 27 | Report which of the following are publicly available and where they can be found: template data collection forms; data extracted from included studies; data used for all analyses; analytic code; any other materials used in the review. | - |

Supplementary Table 2 Search Strategy

| Database | Search Strategy |
| --- | --- |
| MedLine via PubMed | coronavirus[MeSH] OR coronavirus[All Fields] OR covid 2019[All Fields] OR SARS-CoV-2[All Fields] OR SARS-CoV-2[MeSH] OR SARS-CoV-19[All Fields] OR covid-19[All Fields]OR covid-19[MeSH] (Number of studies: 291,436)  AND  vaccine[MeSH] OR vaccination[MeSH] OR vaccine[All Fields] OR vaccination[All Fields] OR immunization[MeSH] OR immunization[All Fields] (Number of studies: 1,602,666)  AND  hematopoietic stem cell transplantation[MeSH] OR hematopoietic stem cell transplantation[All Fields] OR immunotherapy, adoptive[MeSH] OR CAR T[All Fields] OR immunocompromised[All Fields] OR immunosuppressed[All Fields] (Number of studies: 663,539)  (Final number of studies: 2,992) |
| Embase | 'hematopoietic stem cell transplantation'/exp OR 'hematopoietic stem cell transplantation' OR 'adoptive immunotherapy'/exp OR 'chimeric antigen receptor t-cell'/exp OR 'chimeric antigen receptor t-cell' OR 'immunocompromised patient' OR 'immunosuppressive treatment' (Number of studies: 360,530)  AND  'coronavirus infection'/exp OR coronavirus OR 'coronavirus disease 2019'/exp OR (covid AND 2019) OR 'covid 2019' OR 'sars cov 2' OR 'severe acute respiratory syndrome coronavirus 2'/exp (Number of studies: 305,608)  AND  'vaccine'/exp OR vaccine OR 'vaccination'/exp OR vaccination OR 'immunization'/exp OR immunization (Number of studies: 674,254)  (Final number of studies: 2387) |
| Web of Science | **(ALL=(hematopoietic stem cell transplantation) OR ALL=( CAR T)) OR ALL=( immunocompromised) OR ALL=(immunosuppressed)**  (Number of studies: [135,494](https://www.webofscience.com/wos/woscc/summary/1d7b4a38-ef26-4207-bdfa-1c2c0cae4844-43fec813/relevance/1))  AND  **(ALL=(vaccine)) OR ALL=( vaccination)) OR ALL=(immunization)**  (Number of studies: [479,342](https://www.webofscience.com/wos/woscc/summary/4bcbe47f-8be7-4249-8272-35536a510804-43febeae/relevance/1))  AND  **(ALL=(covid-19)) OR ALL=(SARS-CoV-2)**  (Number of studies: [313,833](https://www.webofscience.com/wos/woscc/summary/5d9133a9-60b0-487a-bcf7-0379c411dce9-43feb7c3/relevance/1))  (Final number of studies: 584) |
| MedRxiv and BioRxiv | Search the following keywords in titles and abstracts:  "coronavirus" OR "covid" OR "SARS-CoV-2" OR "SARS-CoV-19" OR "covid-19" (Number of studies: 24207)  AND  "vaccine" OR "vaccination" OR "immunization" OR "immunity" (Number of studies: 13039)  AND  "hsct" OR "cart" OR "stem cell transplantation" OR "immunocompromise" OR "chimeric" (Number of studies: 9224)  (Final number of studies: 122) |

Supplementary Table 3 Risk of bias assessment

| Study | Bias due to confounding | Bias in selection of participants into the study | Bias in classification of interventions | Bias due to deviation from intended interventions | Bias due to missing data | Bias in measurement of outcomes | Bias in selection of the reported results | Overall RoB |
| --- | --- | --- | --- | --- | --- | --- | --- | --- |
| Agnieszka Matkowska-Kocjan | 2 | 1 | 1 | 1 | 1 | 2 | 1 | 2 |
| Alexis Maillard | 2 | 2 | 1 | 1 | 1 | 1 | 1 | 2 |
| Amandine Le Bourgeois | 1 | 2 | 1 | 1 | 1 | 2 | 1 | 2 |
| Amandine Le Bourgeois-2 | 1 | 2 | 1 | 1 | 2 | 1 | 1 | 2 |
| Anne-Claire Mamez | 1 | 2 | 1 | 1 | 2 | 2 | 1 | 2 |
| Binod Dhakal | 2 | 2 | 1 | 1 | 1 | 1 | 1 | 2 |
| Caroline Pabst | 1 | 1 | 1 | 1 | 1 | 1 | 1 | 1 |
| José Luis Piñana | 2 | 2 | 1 | 1 | 1 | 2 | 2 | 2 |
| Kalpana Parvathaneni | 1 | 1 | 1 | 1 | 1 | 2 | 1 | 2 |
| Katie Healy | 2 | 2 | 1 | 1 | 1 | 2 | 1 | 2 |
| Lorenzo Canti | 2 | 1 | 1 | 2 | 1 | 2 | 2 | 2 |
| Lorenzo Canti-2 | 2 | 1 | 1 | 1 | 1 | 1 | 2 | 2 |
| Maciej Majcherek | 2 | 1 | 1 | 1 | 1 | 1 | 1 | 1 |
| Marika Watanabe | 1 | 1 | 1 | 1 | 1 | 2 | 1 | 2 |
| Martina Chiarucci | 2 | 1 | 1 | 1 | 1 | 2 | 1 | 2 |
| Monika Lindemann | 1 | 1 | 1 | 1 | 1 | 1 | 1 | 1 |
| Muhammad Bilal Abid | 2 | 1 | 1 | 1 | 1 | 1 | 1 | 2 |
| Noga Shem-Tov | 1 | 2 | 1 | 1 | 1 | 1 | 1 | 2 |
| Patrice Chevallier | 2 | 1 | 1 | 1 | 1 | 2 | 1 | 2 |
| Peter Bergman | 1 | 2 | 2 | 1 | 1 | 2 | 2 | 2 |
| Rabah Redjoul | 2 | 1 | 1 | 2 | 2 | 2 | 1 | 2 |
| Ron Ram | 2 | 2 | 1 | 1 | 2 | 2 | 2 | 2 |
| Roni Tamari | 1 | 1 | 1 | 1 | 2 | 2 | 2 | 2 |
| Sandra Easdale | 2 | 2 | 1 | 2 | 1 | 2 | 2 | 2 |
| Saurabh Dahiya | 2 | 1 | 2 | 2 | 2 | 1 | 1 | 2 |
| Thomas A. Fox | 2 | 2 | 1 | 1 | 2 | 1 | 3 | 3 |
| Thomas Gastinne | 2 | 2 | 1 | 1 | 1 | 2 | 1 | 2 |

*1 low 2 moderate 3 serious 4 critical

Supplementary Table 4 Safety evaluation after COVID-19 vaccination in patients receiving HSCT or CAR T therapy

|  |  | Systemic adverse effect | | | | | | | | | | | | | | Local adverse effect | | | | | | | | | | | |
| --- | --- | --- | --- | --- | --- | --- | --- | --- | --- | --- | --- | --- | --- | --- | --- | --- | --- | --- | --- | --- | --- | --- | --- | --- | --- | --- | --- |
| Study (N) | Dose | Fever | Fatigue | Headache | Chills | Muscle pain | Joint pain | Vomiting/Nausea/Diarrhea | Axillary lymphadenopathy | | Cytopenia development/exacerbation | | | GVHD exacerbation | | | Pain | | | Swelling | | | Redness | | | Other | |
| Agnieszka Matkowska-Kocjan (65) | 1 | 3 (4.6%) | 10 (15.4%) | 10 (15.4%) | 4 (6.2%) | 10 (15.4%) | 2 (3.1%) | 4 (6.2%) | 2 (3.1%) | | - | | | - | | | 20 (30.8%) | | | 2 (3.1%) | | | - | | | - | |
| Agnieszka Matkowska-Kocjan (58) | 2 | 5 (8.6%) | 15 (25.9%) | 14 (24.1%) | 7 (12.1%) | 14 (24.1%) | 4 (6.9%) | 4 (6.9%) | 1 (1.7%) | | - | | | - | | | 20 (34.5%) | | | 6 (10.3%) | | | 2 (3.4%) | | | Pruitus: 3 (5.2%) | |
| Amandine Le Bourgeois (106) | 1 | Grade I or II adverse reactions: 51 (48.1%) | | | | | | | | | | | | | | | | | | | | | | | | | |
| Amandine Le Bourgeois (87) | 2 | Grade I or II adverse reactions: 34 (39.1%) | | | | | | | | | | | | | | | | | | | | | | | | | |
| Anne-Claire Mamez (63) | 2 | No grade III-IV adverse event | | | | | | | | | | | | | | | | | | | | | | | | | |
| Caroline Pabst (167) | 1 and 2 | - | - | - | - | - | - | - | - | | - | 10 (6.0%) | | | - | | | - | | | - | | | - | | |  |
| José Luis Piñana  （397） | 1 | 4（1%） | - | 6（1.5%） | - | 9（2.3%） | - | - | - | | - | - | | | 31（7.8%） | | | - | | | - | | | 11（2.8%） | | |  |
| José Luis Piñana  （397） | 2 | 6（1.5%） | - | 5（1.3%） | - | 11（2.8%） | - | - | - | | - | - | | | 26（6.5%） | | | - | | | - | | | 9（2.3%） | | |  |
| Lorenzo Canti-2 (40) | 3 | Dyspnea: 1 (2.5%), transverse myelitis: 1 (2.5%) | | | | | | | | | | | | | | | | | | | | | | | | | |
| Maciej Majcherek (93) | 2 | No grade III-IV adverse event; pain, headache, fatigue, chills observed | | | | | | | | | | | | | | | | | | | | | | | | | |
| Marika Watanabe (25) | 1 | 1 (4%) | 5 (20%) | 4 (16%) | 2 (8%) | 3 (12%) | 2 (8%) | 2 (8%) | - | | - | | | - | | | 20 (80%) | | | 2 (8%) | | | 1 (4%) | | | - | |
| Marika Watanabe (25) | 2 | 1 (4%) | 13 (52%) | 7 (28%) | 2 (8%) | 3 (12%) | 1 (4%) | 1 (4%) | - | | - | | | - | | | 17 (68%) | | | 6 (24%) | | | 2 (8%) | | | - | |
| Martina Chiarucci (50) | 2 | Systemic adverse reactions: 2 (4.0%) | | | | | | | | | | | | | | | | | | | | | | | - | | |
| Noga Shem-Tov (152) | 1 | Systemic adverse effect 8 (5.3%) | | | | | | | | | | | | | | - | | | - | | | - | | | - | | |
| Noga Shem-Tov (152) | 2 | Systemic adverse effect 20 (13.2%) | | | | | | | | | | | | | | - | | | - | | | - | | | - | | |
| Patrice Chevallier (94) | 1 | 1 (1.1%) | 19 (20.2%) | 12 (12.8%) | 7 (7.4%) | 7 (7.4%) | - | 2 (2.1%) | - | | - | | | - | | | 19 (20.2%) | | | 6 (6.4%) | | | 5 (5.3%) | | | - | |
| Peter Bergman (90) | 2 | 2 (2.2%) moderate, 3 (3.3%) severe adverse reactions | | | | | | | | | | | | | | | | | | | | | | | | | |
| Rom Ram (80) | 1 | Non-hematologic grade I or II adverse reactions: 11 (13.8%) | | | | | | | | 10 (12.5%) | | | 3 (4.5%) | | | - | | | - | | | - | | | - | | |
| Rom Ram (80) | 2 | Non-hematologic grade I or II adverse reactions: 18 (23.5%) | | | | | | | | 8 (10%) | | | 4 (4.5%) | | | - | | | - | | | - | | | - | | |
| Thomas Gastinne (20) | 1 | 1 (5%) | - | 1 (5%) | 1 (5%) | - | - | - | - | | - | | | - | | | 3 (15%) | | | - | | | 1 (5%) | | | - | |
| Thomas Gastinne (20) | 2 | - | 1 (5%) | - | - | - | - | - | - | | - | | | - | | | 1 (7.1%) | | | - | | | 1 (7.1%) | | | - | |

Supplementary Table 5 Impact factors of seroconversion rate in HSCT or CAR T recipients

| Study | Time to vaccination post-HSCT/CAR T | | The ongoing immunosuppressive treatment | | Number of lymphocytes before vaccination | | Chronic graft-versus-host disease (cGVHD) before vaccination | |
| --- | --- | --- | --- | --- | --- | --- | --- | --- |
|  | Details of factor and seroconversion | Correlation | Details of factor and seroconversion | Correlation | Details of factor and seroconversion | Correlation | Details of factor and seroconversion | Correlation |
| Agnieszka Matkowska-Kocjan  (Allo-HSCT) | 3-27 years (median 10.5) | Not significant  (due to low absolute value of R^2^)  p=0.02,  R^2^= -0.09250 | 1-20 years (median 7.5) from the end of immunosuppression, 4 patients still receiving treatment | Not significant | Within normal range in 57 patients, below normal range in 8 patients | Not significant | 15 patients showed symptoms of cGVHD at vaccination | Not significant |
| Alexis Maillard  (After 2 doses, Allo-HSCT) | < 12 months:  R/N 67/77  ≥ 12 months:  R/N 471/72 | Significant correlation (p<0.001) | Ongoing treatment (antineoplastic/ immunosuppressive treatments/ rituximab):  R/N 167/139 | Significant correlation  (Ongoing antineoplastic treatments: p=0.015  Ongoing immunosuppressive treatments  P<0.001  Rituximab  P<0.001) | Absolute lymphocyte count (G/L):  ≥ 1G/L R/N 370/51  < 1G/L R/N 94/85  CD4 T cell count:  ≥ 500/mm3 R/N 160/14  < 500/mm3 R/N 225/92  B cells:  ≥ 100/mm3 R/N 250/24  < 100/mm3 R/N 43/60 | Significant correlation  (Absolute lymphocyte count:  p<0.001  CD4^+^ T cells:  p=0.001  B cells:  p<0.001) | History of GVHD requiring systemic treatment:  R/N 188/51 | Not significant (p=1) |
| Amandine Le Bourgeois  (Allo-HSCT) | 654 days (91-6198);  After 1 dose: <12 mo (R/N 4/25), 12-24mo (R/N 18/18), >24mon (R/N 41/11);  After 2 doses: <12 mo (R/N 15/14), 12-24mo (R/N 32/4), >24mon (R/N 50/2) | Significant correlation  (After 1 dose: p=0.001  After 2 doses: p<0.001) | 32 total patients had ongoing treatment for relapse or relapse prevention: 13 corticosteroids, 3 ruxolitinib, 10 cyclosporine A, 6 chemotherapy.  After 1 dose: no treatment (R/N 53/32), on treatment (R/N 10/22)  After 2 doses: no treatment (R/N 77/8), on treatment (R/N 20/12) | Significant correlation  (After 1 dose: p=0.005  After 2 doses: p<0.001) | Median 1400 (150-9880 cells/ul).  After 1 dose:  ≥1000 (R/N 54/27), <1000 (R/N 9/27);  After 2 doses: ≥1000 (R/N 74/7), <1000 (R/N 23/13) | Significant correlation  (After 1 dose: p<0.001  After 2 doses: p<0.001) | After 1 dose:  Previous GVHD (R/N 32/30), no previous GVHD (R/N 31/24);  After 2 doses: Previous GVHD (R/N 51/11), no previous GVHD (R/N 46/9) | Not significant (After 1 dose: p=0.74  After 2 doses: p=0.87) |
| Amandine Le Bourgeois-2  (Allo-HSCT) | 719 (91–6198) days | Not reported | Ongoing treatment (chemotherapy or immunosuppressive drugs): 19 patients  No treatment: 61 patients | Not reported | Lymphocyte count at Dose 1, ×10^9 /l, median (range): 1.390 (0.260–9.880) | Not reported | 44 patients had previous GVHD, 36 patients didn’t have GVHD | Not reported |
| Anne-Claire Mamez  (Allo-HSCT) | 14 (3-150) months:  <6 months R/N 6/7;  >6 months R/N 42/8 | Significant correlation  (p=0.0016) | ATG treatment (R/N): 27/14 | Significant correlation  (p=0.004) | Lymphocyte counts >1 G/L R/N 37/9;  Lymphocyte counts >1 G/L R/N 12/5 | Significant correlation  (p=0.02) | Not reported | Not reported |
| Binod Dhakal  (Allo-HSCT; Auto-HSCT; CAR T) | Auto-HSCT: R/N (median time, months)  30 (3-173)/ 30 (2-96);  Allo-HSCT: R/N: 26 (4-154)/ 25 (3-155);  CAR T: R/N: 24 (8-31)/ 6 (3-37) | Not significant  (Auto-HSCT: p=0.50  Allo-HSCT: p=0.68  CAR T: p=0.09) | Auto-HSCT: 12 patients (9 lenalidomide ± other drugs，2 rituximab, 1 nivolumab)  Allo-HSCT: 46 patients (16/6 ruxolitinib ± other drugs, 5 responders sirolimus ± other drugs, 3/4 mycophenolate moefetil, 2/3 tacrolimus, 2/1 prednisone, 1 responder ibrutinib, ohters)  CAR T: (-) | Not significant  (Auto-HSCT: on treatment (R/N 8/4), p=0.58  Allo-HSCT: no treatment (R/N 18/7), on treatment (R/N 31/15). P=0.69  CAR T:(-)) | Allo-HSCT:  Median (range) CD4 T cell count: 327 (44-1165)/ 274 (56- 576);  Median (range) CD8 T cell count: 278 (46-1739)/ 276 (34-1440) | Not significant  (Allo-HSCT: CD4^+^ T cells: p=0.10;  CD8^+^ T cells: p=0.73) | Allo-HSCT: 35 patients had active cGVHD (R/N 23/12) | Not significant  (p=0.55) |
| Caroline Pabst  (Allo-HSCT) | 1215 (106–9111) | Not significant  (All data, index > 100: OR (95%CI): 0.677 (0.163-2.815), p=0.591  No IS, index > 20: OR (95%CI): 0.631 (0.083-4.771), p=0.656  No IS, index > 20: OR (95%CI): 3.730 (0.620-22.448), p=0.150) | No immunosuppression: 107 patients, on immunosuppression/maintenance therapy: 60 patients | Significant correlation  (All data, index > 100: OR (95%CI) ≥2 IS drugs: 0.026 (0.002-0.294), p=0.003) | All data, index > 100: OR (95%CI) B cell counts: 3.291 (1.122- 9.649) CD4^+^ T cell counts: 1.837 (0.403 8.369) | Significant correlation  (B cells:  P=0.030  CD4^+^ T cells:  P=0.432) | Not reported | Not reported |
| José Luis Piñana  (Allo-HSCT;  Auto-HSCT) | Allo-HSCT: 98 (4–646) months;  Auto-HSCT:  88 (3–763) | Significant correlation  OR (95%CI)  Allo-HSCT: <6 months 0.05 (0.006–0.43), p=0.008; 6months-1 year: 0.4 (0.14–1.7), p=0.2; ≥1 year, 1, p=/; <1 year, 0.24 (0.094–0.65), p=0.005  Auto-HSCT:  <6 months 1 (0.11–9.2), p=0.9; 6months-1 year: 0.33 (0.028–4.1), p=0.4; ≥1 year, 1, p=/; <1 year, 0.72 (0.13–3.86), p=0.7 | Allo-HSCT:  GVHD prophylaxis: 140 posttransplant cyclophosphamide, 116 Sirolimus, 229 calcineurin inhibitor  Immunosuppressors: 104 patients; Immunosuppressors without corticosteroids: 86 patients; Corticosteroids 19 (< 0.5 mg/kg 15, ≥ 0.5 mg/kg 4)  Auto-GVHD:  Immunosuppressors: 45 patients; Immunosuppressors without corticosteroids: 18 patients; Corticosteroids 28 (< 0.5 mg/kg 3, ≥ 0.5 mg/kg 25) | Significant correlation Allo-HSCT:  GVHD prophylaxis: posttransplant cyclophosphamide 1.2 (0.69–2.09), p=0.5, Sirolimus 1.1 (0.64–2.03), p=0.62, calcineurin inhibitor 1, p=/.  Immunosuppressors: 0.4 (0.24–0.75), p=0.003;  Immunosuppressors and corticosteroids: 1, p=/; Immunosuppressors without corticosteroids: 1.7 (0.6–4.8), p=0.3; none of them: 2.8 (1.03–7.6), p=0.04. | Allo-HSCT: Absolute neutrophile counts, median (range) (×10^9 /ml): 2.96 (0.06–11.57); Absolute lymphocyte counts, median (range) 2.15 (0.28–19.4).  Auto-HSCT: Absolute neutrophile counts, median (range) (×10^9 /ml): 2.7 (0.44–15.4); Absolute lymphocyte counts, median (range) 1.53 (0.65–4.1) | Significant correlation  Allo-HSCT:  Lymphocyte count < 0.5 × 10^9/ml: 0.1 (0.03–0.33), p<0.0001; Lymphocyte count < 1.0 × 10^9/ml: 0.25 (0.12–0.4), p<0.0001.  Auto-HSCT:  Lymphocyte count < 1.0 × 10^9/ml: 0.5 (0.12–2.28), p=0.39. | Allo-HSCT:  cGVHD 82 patients | Not significant Active GvHD at vaccination (including cGVHD and acute GVHD): 0.56 (0.3–1.03), p=0.06 |
| Kalpana Parvathaneni  (CAR T) | 22 (3-126) months | Not reported | Current chemotherapy and/or immunotherapy  None: 11 patients  Ponatinib: 1 patient | Not reported | Mean B-cell count/μL [SD] for RBD IgG positive, 57.2 [20.2]; for RBD-IgGnegative,9[10.1] | Significant correlation (p<0.05) | Not reported | Not reported |
| Katie Healy  (Allo-HSCT/  CAR T) | Not reported | Not reported | 9 patients: Corticosteroids;  18 patients: other immunosuppressants | Not reported | Absolute lymphocyte count (x10^9/L): 1.3 (0.88-1.70) | Not reported | Not reported | Not reported |
| Lorenzo Canti  (Allo-HSCT) | 31 months (6-57) | Significant correlation  Anti-RBD Ab response:  univariate p=0.88, OR 1.11 (0.28-4.48), v p=0.28, OR 4.01 (0.32-50.5).  Anti-RBD Ab titer: univariate p=0.013, estimate 0.99 (SE 0.63), multivariate p=0.052, estimate 0.80 (SE 0.40).  Detectable neutralizing antibodies:  univariate p=0.052, OR 3.33 (0.99-11.2), multivariate p=0.048, OR 5.72 (1.02-32.2). | 5 Tacrolimus, 1 Photopheresis, 1 Photopheresis + methyl-prednisolone < 32 mg/day, 1 mycophenolate mofetil, 1 mycophenolate mofetil + methyl-prednisolone < 32 mg/day, 1 sirolimus, 2 sirolimus + methyl-prednisolone < 32 mg/day, 2 Photopheresis + ruxolitinib | Not reported | Anti-RBD Ab levels: Naïve CD4 T cells p=0.0005, TFH p=0.015, naïve B cells p= 0.011, class-switched memory B cells p< 0.0001, class-unswitched memory B cells p<0.0001.  NT50 Abs: Naïve CD4 T cells p=0.001, TFH p=0.0625, naïve B cells p= 0.0267, class-switched memory B cells p< 0.0001, class-unswitched memory B cells p<0.0001 | Significant correlation | 29 never/only mild, 2 priors moderate/severe solved*, 9 ongoing moderate/severe | Significant correlation  Anti-RBD Ab response:  univariate p=0.0080, OR 0.014 (<0.001-0.33), multivariate p=0.018, OR 0.014 (<0.001-0.49).  Anti-RBD Ab titer: univariate p<0.0001, estimate -4.00 (SE 0.78), multivariate p<0.0001, estimate -3.87 (SE 0.66). |
| Lorenzo Canti-2  (Allo-HSCT) | 31 (6-58) months | Not reported | No systemic immunosuppression 24 patients, on immunosuppression 14 patients | Not reported | There was a correlation between absolute counts of unswitched memory B cells (r = 0.51, p = 0.001), class-switched memory B cells (r = 0.40, p = 0.01), naive B cells (r = 0.35, p = 0.03), and TFH (r = 0.37, p = 0.027) | Significant correlation | Never/only mild 27 patients; Prior moderate/severe solved 1 patient; Ongoing moderate / severe 10 patients | Not significant  (p < 0.05) |
| Maciej Majcherek  (Allo-HSCT;  Auto-HSCT) | Auto-HSCT:  10 (4–38) months (No specific R/N data shown)  Allo-HSCT: R/N  27.3 (4.9–111.8)/ 7.7 (3.3–15.8) | Significant correlation  (Allo-HSCT:  p=0.02) | Immunosuppressive treatment:  Yes R/N 12/6  No R/N 44/1  Calcineurin inhibitor:  Yes R/N 4/6;  No R/N 52/1 | Significant correlation  (Immunosuppressive treatment:  p<0.001;  Calcineurin inhibitor:  p<0.001) | T cells (G/L)  : R/N  0.87 (0.23–5.86)/1.03 (0.42–2.89);  B cells: R/N 0.22 (0.0–1.11)/ 0.07 (0.0–0.35);  CD4 T cells: R/N 0.22 (0.04–0.73)/ 0.21 (0.09–0.64)  CD8 T cells: R/N 0.57 (0.11–5.34)/ 0.64 (0.24–2.21);  NK cells: R/N 0.12 (0.02–0.71)/ 0.17 (0.12–0.37) | Significant correlation  (T cells:  p=0.83  B cells:  p=0.038  CD4 T cells:  p=0.64  CD8 T cells:  p=0.72  NK cells:  p=0.10) | Active acute or chronic cGVHD at vaccination:  Yes R/N 7/5;  No R/N 49/2 | Significant correlation  (p<0.001) |
| Martina Chiarucci  (Allo-HSCT;  Auto-HSCT) | 369 days (5-736) | Not reported | 6 allo-HSCT patients received immunosuppressive therapy: 4 cyclosporine (less than 50 mg/day), 2 low doses of systemic steroid therapy alone (prednisone <0.5mg/kg/ day) | Significant correlation  (On treatment: R/N 1/5; no treatment R/N 5/1. p=0.0003) | R/N:  B CD19+/mmc: ≤100 (11/6), >100 (21/2); T CD4+/mmc: ≤200 (12/5), >200 (20/6); T CD8+/mmc: ≤300 (10/2), >300 (22/9); NK CD56+/mmc: ≤100 (4/6), >100 (27/4) | Significant correlation  (B cell: p=0.0003; CD4^+^ T cells: p=0.07;  CD8^+^ T cells: p=0.001,  NK cells: p<0.0001) | 5 allo-HSCT patients had GVHD, with GVHD: R/N 3/2; no GVHD: R/N 3/4. | Not significant (p=0.16) |
| Marika Watanabe  (Allo-HSCT) | 1605 days (163–4126 days | Significant correlation  <1 year: R/N 2/2  >1 year: R/N 17/4 | On immunosuppression: 7  No immunosuprression: 18 | Significant correlation (Steroids yes/no:  p=0.01  Calcineurin inhibitors yes/no:  p=0.45) | Absolute lymphocyte counts:  >1000/µL, 19 patients;  <1000/µL,  6 patients | Significant correlation  (>1000/µL yes/no: p=0.01) | Not reported | Not reported |
| Monika Lindemann  (Allo-HSCT) | 30 months (5-391 months) | Significant correlation  IgG level: r = 0.31, p = 0.0006 | 57 patients received immunosuppressive drugs at the time of vaccination | Not reported | Not reported | Not reported | Not reported | Not reported |
| Muhammad Bilal Abid  (Allo-HSCT;  Auto-HSCT;  CAR T) | “We found no significant differences in  seroconversion based on age, interval between immunotherapy and vaccination,  corticosteroid usage, active graft versus  host disease (in alloHCT), immunosuppression status (including active immunosuppressant use), disease relapse prior  to booster, absolute lymphocyte count  (ALC), or CD4, CD8, or IgG levels.” | Not significant | / | Not significant | / | Not significant | / | Not significant |
| Noga Shem-Tov  (Allo-HSCT) | Median (IQR) years R/N:  3.6 (2.1–6.5)/ 3.1 (1.9–5.5) | Significant correlation Overall: p=0.41  6-12 months: R/N 1/4, p=0.0145  12-24 months:  R/N 28/6  >24 months:  89/24 | 63 no therapy, 32 ciclosporin, 64 Prednisone, 8 mycophenolate | Significant correlation  No therapy: R/N 59/4, p <0.0001;  Ciclosporin: R/N 16/16, p <0.0001; Prednisone: R/N 43/21, p=0.084;  Mycophenolate: R/N 1/7, p <0.0001 | Lymphocyte absolute (K/μl): R/N 2.52 ± 1.37/2.25 ± 1.67 | Not significant (p=0.35) | None cGVHD: R/N 72/13;  Mild cGVHD: R/N 31/8;  Moderate-severe cGVHD: R/N 13/15 | Significant correlation (p=0.002) |
| Patrice Chevallier  (Allo-HSCT) | Median 22.1 months R/N:  (3–206):  <12mo (5/24), 12-24mo (19/16), >24mo (38/10) | Significant correlation (p<0.0001) | 31 patients had ongoing immunosuppressive treatment for relapse or relapse prevention: 13 corticosteroids, 3 ruxolitinib, 9 cyclosporine A, 6 chemotherapy (venetoclax +ibrutinib n = 1; methotrexate n = 1; tafasitamab+ revlimid n = 1, 3 AML 5′azacytidine) | Significant correlation  No treatment: R/N 53/28; on treatment: R/N 9/22. p=0.001 | Lymphocyte count (×10^9 /L): 1.4 (0.15–9.9) | Significant correlation <1×10^9 /L: R/N 8/26;  >1×10^9 /L: R/N 54/28. p<0.001 | 57 patients showed previous GVHD.  With previous GVHD: R/N 29/28; no GVHD: R/N 33/22 | Not significant |
| Peter Bergman  (Allo-HSCT;  CAR T) | Early <6 months (n=10); Intermediate 6 - 12 months (n=12); Late >12 months (n=65) | Significant correlation  R/N:  Early: 3/1, p=0.02, OR 6·25 (1·26-31·9); intermediate 8/4, p=0.26, OR 4·17 (0·18-43·0); late 50/4. | 13 patients received corticosteroids, 27 patients received other immunosuppressive agents | Not reported | Absolute lymphocyte count (×10^9 /L): 1.6 (0.3-7.0) | Not reported | Severe cGVHD was identified as a risk factor for failure to seroconvert. | Significant correlation  p=0.02, OR (CI) 8 (1·43-49·82) |
| Rabah Redjoul  (Allo-HSCT) | 23 months (3-213) | Significant correlation | Systemic immunosuppressive treatments within 3 months of vaccination | Significant correlation  Patients with IgG(S-RBD) titres above (n=52) and below (n=36) 4160 AU/mL, systemic immunosuppression within 3 months before vaccination 2, n (%): 9 (17%) / 29 (81%), p<0.001 | Patients with IgG(S-RBD) titres above (n=52) and below (n=36) 4160 AU/mL, Lymphocyte count in PB, n (%) (Above/Below): < 1G/L: 3 (6%) / 15 (42%), ≥ 1G/L: 49 (94%) / 21(58%) | Significant correlation  (p<0.001) | Patients with IgG(S-RBD) titres above (n=52) and below (n=36) 4160 AU/mL, history of GVHD requiring systemic treatment, n (%) (Above/Below): 22 (61%) / 22 (42%) | Not significant  (p=0.13) |
| Ron Ram  (Allo-HSCT;  CAR T) | Allo-HCT: 32 months (3-263); CAR T: 9 months (3-17) | Significant correlation  (univariate: p=0.012; multivariate p=0.032) | 6 on low-intensity immunosuppressive therapy, 32 on high-intensity immunosuppressive therapy (prednisone dose of ≥0.25 mg/kg/day or another IST medication.); 6 on active chemotherapy. | Not significant  (Low-intensity immunosuppressive therapy: p=0.563) | Total CD19 + lymphocyte count: 89 (0-1078);  Total CD4 + lymphocyte: 325 (10-1575); Total CD8 + lymphocyte: 756 (48-4158); CD4 + /CD8 + ratio: 0.48 (0.14-2.9) | Significant correlation  (CD19 cells: univariate: p=0.012; multivariate: p=0.047) | 40 patients exhibited active cGVHD and 3 patients exhibited previous non-active cGVHD. | Not significant  (Active GVHD: p=0.467) |
| Roni Tamari  (Allo-HSCT;  Auto-HSCT;  CAR T) | 1007 days (63-7026) | Significant correlation  (Allo-HCT: p=0.282;  Auto-HCT: p=0.001) | 46 on immune suppression with tacrolimus/ cyclosporine or ruxolitinib (among allo-HCT only); 21 on steroids; 13 on Daratumumab; 20 on IMiD; 11 on other chemotherapy | Significant correlation  R/N (spike IgG)  Allo-HSCT: on TCR (41/5), no TCR (92/11), p>0.99; On steroids (14/7), no steroids (119/9), p=0.002.  Auto-HSCT: on IMiDs (18/2), no IMiDs (35/6), p>0.99; On Dara (11/1), no Dara (42/7), p>0.99.  R/N (neutralizing abs):  Allo-HSCT: on TCR (27/13), no TCR (71/13), p=0.036; On steroids (7/9), no steroids (91/17), p=0.001.  Auto-HSCT: on IMiDs (13/6), no IMiDs (27/6), p=0.317; On Dara (7/4), no Dara (33/8), p=0.253. | R/N (spike IgG):  Allo-HSCT: CD4 count: <200 (15/6), ≥200 (75/6), p=0.016; CD19 count: <50 (6/7), ≥50 (84/5), p<0.001.  Auto-HSCT: CD4 count: <200 (4/2), ≥200 (18/5), p=0.612; CD19 count: <50 (3/6), ≥50 (19/1), p=0.001 | Significant correlation  R/N (neutralizing abs)  Allo-HSCT: CD4 count: <200 (9/7), ≥200 (60/11), p=0.019; CD19 count: <50 (2/8), ≥50 (67/10), p<0.001.  Auto-HSCT: CD4 count: <200 (2/2), ≥200 (14/5), p=0.557; CD19 count: <50 (3/3), ≥50 (13/4), p=0.318 | Not reported | Not reported |
| Sandra Easdale  (Allo-HSCT) | R/N:  3-6 months (1/7); >6 months (20/27) | Not significant  (p=0.136) | 29 patients on immune suppression | Significant correlation  (On treatment: R/N 6/23, no treatment R/N 15/11, p=0.0062) | Lymphocyte count at time of vaccination, ×10^9 /L, median: R/N 1.33/1.11 | Not significant  (p=0.062) | 35 patients exhibited cGVHD. R/N 11/24 | Not significant  (p=0.173) |
| Saurabh Dahiya  (CAR T) | 13 patients (median 33 days after CAR T; range, 24-447);  3 allo-HSCT + CAR T patients: (median, 499 days after CAR T cells; range, 466-532) | Not reported | All patient had anti-CD 20 therapy and time from last anti-CD20 therapy to 1st vaccine, median (range): 532.5 (84-3741) days | Not reported | CD4 absolute count: 62 (35-626) cells/uL | Not reported | Not reported | Not reported |
| Thomas A. Fox  (CAR T) | Not reported | Not reported | Not reported | Not reported | Not reported | Not reported | Not reported | Not reported |
| Thomas Gastinne  (CAR T) | Range: 113–819 days, median 401 days. | Not significant | Three patients were still on therapy (revlimid n = 1, tafasitamab n = 1, chemotherapy n = 1) | Not reported | Not reported | Not reported | Not reported | Not reported |

R/N, responders/non-responders. Auto-HSCT, autologous haematopoietic stem cell transplant. Allo-HSCT, allogeneic haematopoietic stem cell transplant. CAR T, chimeric antigen receptor T cell therapy. Anti-RBD Ab, anti‑receptor‑binding domain SARS‑CoV‑2 IgG antibody. NT50: neutralizing antibody titer that reduced the number of infected well by SARS‑CoV‑2 wild type by 50%. IMiD, immunomodulatory drug. Dara, daratumumab.


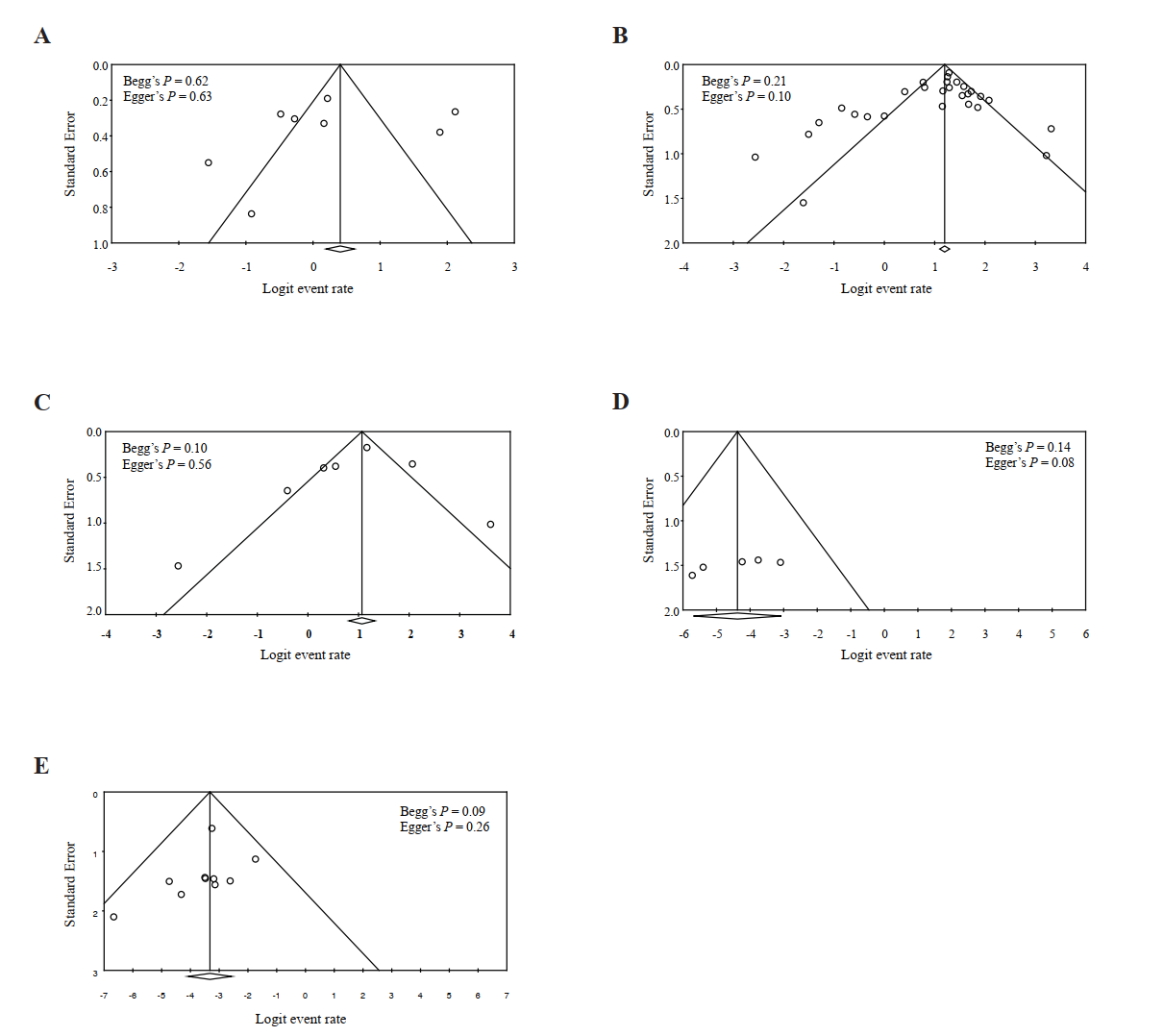


Supplementary Figure 1 Funnel plots

Funnel plot analysis of studies in the meta-analysis. (A) Serologic response after one dose of vaccine. (B) Serologic response after two doses of vaccine. (C) Serologic response after three doses of vaccine. (D) Comparison of seropositive rate in recipients and healthy controls after 1 dose of vaccine. (E) Comparison of seropositive rate in recipients and healthy controls after 2 doses of vaccine.


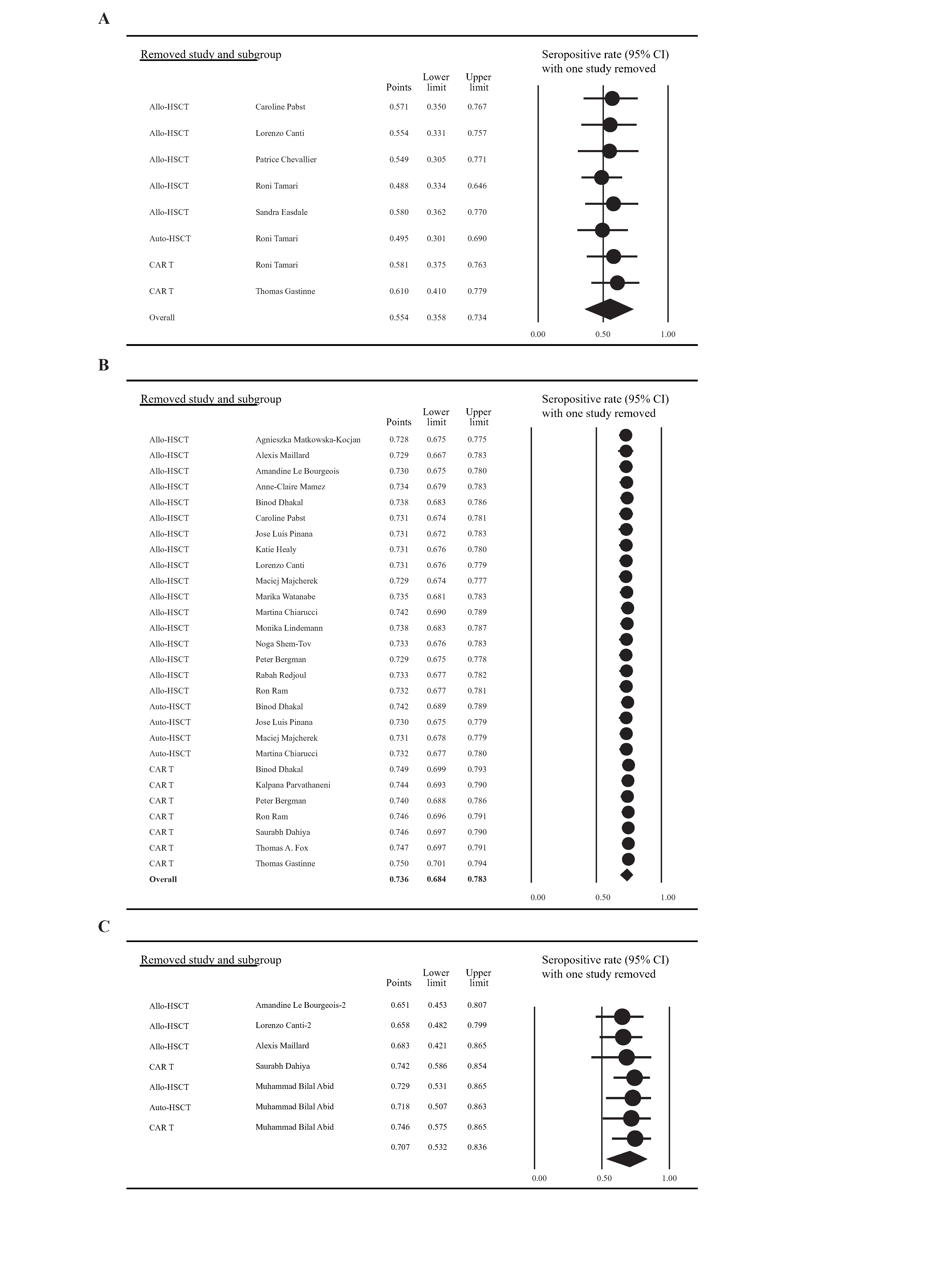


Supplementary Figure 2 Sensitivity analysis excluding one subgroup within a study at a time for “serologic response after COVID-19 vaccination”

(A) Sensitivity analysis for serologic response after one dose of vaccine. (B) Sensitivity analysis for serologic response after two doses of vaccine. (C) Sensitivity analysis for serologic response after three doses of vaccine. The size of the solid circles denotes the mean difference, and the horizontal lines represent the 95% CIs. The diamond denotes the pooled estimate, and the lateral tips of the diamond indicate the 95% CIs.


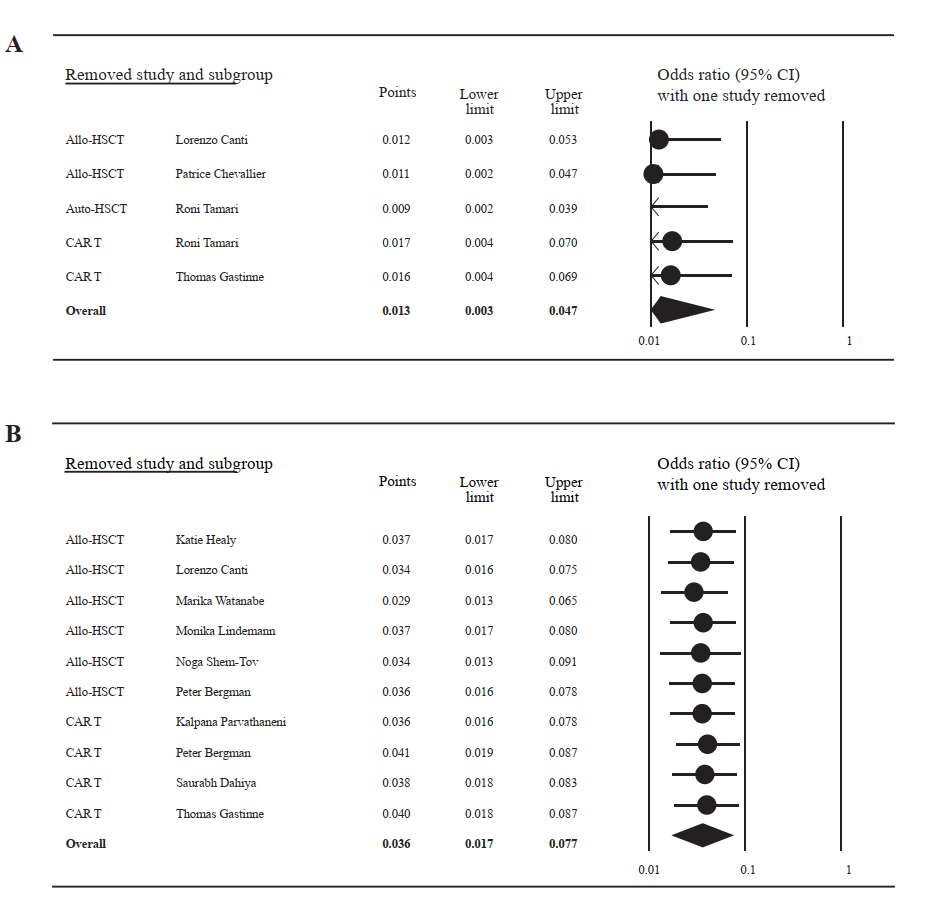


Supplementary Figure 3 Sensitivity analysis excluding one subgroup within a study at a time for “comparison of patients receiving HSCT or CAR T therapy with healthy controls”

(A) Sensitivity analysis for comparison of serologic response after one dose of vaccine. (B) Sensitivity analysis for comparison of serologic response after two doses of vaccine. The size of the solid circles denotes the mean difference, and the horizontal lines represent the 95% CIs. The diamond denotes the pooled estimate, and the lateral tips of the diamond indicate the 95% CIs.


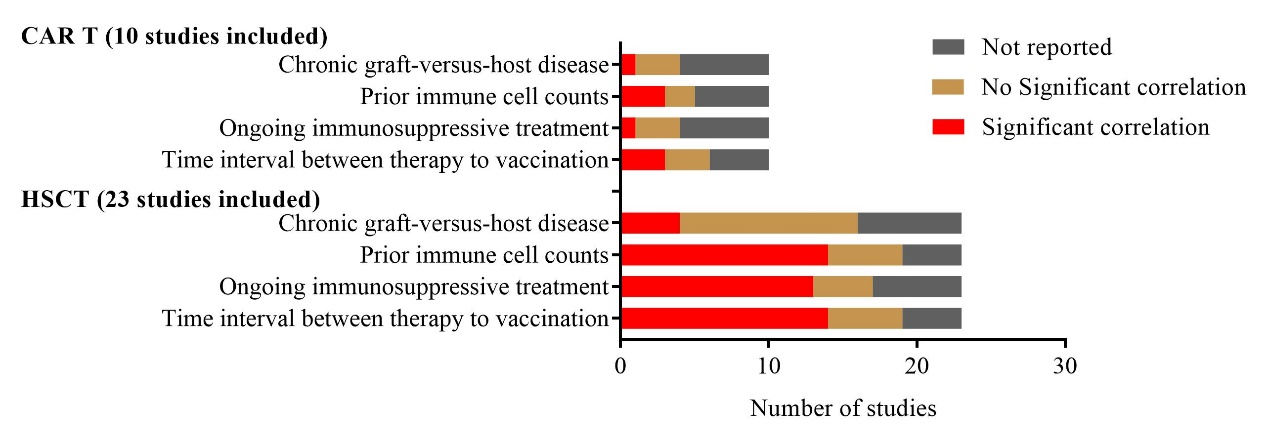


Supplementary Figure 4 Impact factors of seroconversion in HSCT or CAR T-cell recipients

The impact factors of seroconversion in HSCT or CAR T-cell recipients were summarized in four aspacts as annotated in the figure. They were divided into three categories (significant correlation, no significant correlation, not reported) based on the correlation with seroconversion.
